# Measurement of the ${\varvec{t\bar{t}Z}}$ and ${\varvec{t\bar{t}W}}$ production cross sections in multilepton final states using 3.2 fb$^{-1}$ of $\varvec{pp}$ collisions at $\sqrt{s}$ = 13 TeV with the ATLAS detector

**DOI:** 10.1140/epjc/s10052-016-4574-y

**Published:** 2017-01-20

**Authors:** M. Aaboud, G. Aad, B. Abbott, J. Abdallah, O. Abdinov, B. Abeloos, R. Aben, O. S. AbouZeid, N. L. Abraham, H. Abramowicz, H. Abreu, R. Abreu, Y. Abulaiti, B. S. Acharya, L. Adamczyk, D. L. Adams, J. Adelman, S. Adomeit, T. Adye, A. A. Affolder, T. Agatonovic-Jovin, J. Agricola, J. A. Aguilar-Saavedra, S. P. Ahlen, F. Ahmadov, G. Aielli, H. Akerstedt, T. P. A. Åkesson, A. V. Akimov, G. L. Alberghi, J. Albert, S. Albrand, M. J. Alconada Verzini, M. Aleksa, I. N. Aleksandrov, C. Alexa, G. Alexander, T. Alexopoulos, M. Alhroob, B. Ali, M. Aliev, G. Alimonti, J. Alison, S. P. Alkire, B. M. M. Allbrooke, B. W. Allen, P. P. Allport, A. Aloisio, A. Alonso, F. Alonso, C. Alpigiani, M. Alstaty, B. Alvarez Gonzalez, D. Álvarez Piqueras, M. G. Alviggi, B. T. Amadio, K. Amako, Y. Amaral Coutinho, C. Amelung, D. Amidei, S. P. Amor Dos Santos, A. Amorim, S. Amoroso, G. Amundsen, C. Anastopoulos, L. S. Ancu, N. Andari, T. Andeen, C. F. Anders, G. Anders, J. K. Anders, K. J. Anderson, A. Andreazza, V. Andrei, S. Angelidakis, I. Angelozzi, P. Anger, A. Angerami, F. Anghinolfi, A. V. Anisenkov, N. Anjos, A. Annovi, C. Antel, M. Antonelli, A. Antonov, F. Anulli, M. Aoki, L. Aperio Bella, G. Arabidze, Y. Arai, J. P. Araque, A. T. H. Arce, F. A. Arduh, J-F. Arguin, S. Argyropoulos, M. Arik, A. J. Armbruster, L. J. Armitage, O. Arnaez, H. Arnold, M. Arratia, O. Arslan, A. Artamonov, G. Artoni, S. Artz, S. Asai, N. Asbah, A. Ashkenazi, B. Åsman, L. Asquith, K. Assamagan, R. Astalos, M. Atkinson, N. B. Atlay, K. Augsten, G. Avolio, B. Axen, M. K. Ayoub, G. Azuelos, M. A. Baak, A. E. Baas, M. J. Baca, H. Bachacou, K. Bachas, M. Backes, M. Backhaus, P. Bagiacchi, P. Bagnaia, Y. Bai, J. T. Baines, O. K. Baker, E. M. Baldin, P. Balek, T. Balestri, F. Balli, W. K. Balunas, E. Banas, Sw. Banerjee, A. A. E. Bannoura, L. Barak, E. L. Barberio, D. Barberis, M. Barbero, T. Barillari, T. Barklow, N. Barlow, S. L. Barnes, B. M. Barnett, R. M. Barnett, Z. Barnovska-Blenessy, A. Baroncelli, G. Barone, A. J. Barr, L. Barranco Navarro, F. Barreiro, J. Barreiro Guimarães da Costa, R. Bartoldus, A. E. Barton, P. Bartos, A. Basalaev, A. Bassalat, R. L. Bates, S. J. Batista, J. R. Batley, M. Battaglia, M. Bauce, F. Bauer, H. S. Bawa, J. B. Beacham, M. D. Beattie, T. Beau, P. H. Beauchemin, P. Bechtle, H. P. Beck, K. Becker, M. Becker, M. Beckingham, C. Becot, A. J. Beddall, A. Beddall, V. A. Bednyakov, M. Bedognetti, C. P. Bee, L. J. Beemster, T. A. Beermann, M. Begel, J. K. Behr, C. Belanger-Champagne, A. S. Bell, G. Bella, L. Bellagamba, A. Bellerive, M. Bellomo, K. Belotskiy, O. Beltramello, N. L. Belyaev, O. Benary, D. Benchekroun, M. Bender, K. Bendtz, N. Benekos, Y. Benhammou, E. Benhar Noccioli, J. Benitez, D. P. Benjamin, J. R. Bensinger, S. Bentvelsen, L. Beresford, M. Beretta, D. Berge, E. Bergeaas Kuutmann, N. Berger, J. Beringer, S. Berlendis, N. R. Bernard, C. Bernius, F. U. Bernlochner, T. Berry, P. Berta, C. Bertella, G. Bertoli, F. Bertolucci, I. A. Bertram, C. Bertsche, D. Bertsche, G. J. Besjes, O. Bessidskaia Bylund, M. Bessner, N. Besson, C. Betancourt, S. Bethke, A. J. Bevan, W. Bhimji, R. M. Bianchi, L. Bianchini, M. Bianco, O. Biebel, D. Biedermann, R. Bielski, N. V. Biesuz, M. Biglietti, J. Bilbao De Mendizabal, H. Bilokon, M. Bindi, S. Binet, A. Bingul, C. Bini, S. Biondi, D. M. Bjergaard, C. W. Black, J. E. Black, K. M. Black, D. Blackburn, R. E. Blair, J. -B. Blanchard, J. E. Blanco, T. Blazek, I. Bloch, C. Blocker, W. Blum, U. Blumenschein, S. Blunier, G. J. Bobbink, V. S. Bobrovnikov, S. S. Bocchetta, A. Bocci, C. Bock, M. Boehler, D. Boerner, J. A. Bogaerts, D. Bogavac, A. G. Bogdanchikov, C. Bohm, V. Boisvert, P. Bokan, T. Bold, A. S. Boldyrev, M. Bomben, M. Bona, M. Boonekamp, A. Borisov, G. Borissov, J. Bortfeldt, D. Bortoletto, V. Bortolotto, K. Bos, D. Boscherini, M. Bosman, J. D. Bossio Sola, J. Boudreau, J. Bouffard, E. V. Bouhova-Thacker, D. Boumediene, C. Bourdarios, S. K. Boutle, A. Boveia, J. Boyd, I. R. Boyko, J. Bracinik, A. Brandt, G. Brandt, O. Brandt, U. Bratzler, B. Brau, J. E. Brau, H. M. Braun, W. D. Breaden Madden, K. Brendlinger, A. J. Brennan, L. Brenner, R. Brenner, S. Bressler, T. M. Bristow, D. Britton, D. Britzger, F. M. Brochu, I. Brock, R. Brock, G. Brooijmans, T. Brooks, W. K. Brooks, J. Brosamer, E. Brost, J. H Broughton, P. A. Bruckman de Renstrom, D. Bruncko, R. Bruneliere, A. Bruni, G. Bruni, L. S. Bruni, BH Brunt, M. Bruschi, N. Bruscino, P. Bryant, L. Bryngemark, T. Buanes, Q. Buat, P. Buchholz, A. G. Buckley, I. A. Budagov, F. Buehrer, M. K. Bugge, O. Bulekov, D. Bullock, H. Burckhart, S. Burdin, C. D. Burgard, B. Burghgrave, K. Burka, S. Burke, I. Burmeister, J. T. P. Burr, E. Busato, D. Büscher, V. Büscher, P. Bussey, J. M. Butler, C. M. Buttar, J. M. Butterworth, P. Butti, W. Buttinger, A. Buzatu, A. R. Buzykaev, S. Cabrera Urbán, D. Caforio, V. M. Cairo, O. Cakir, N. Calace, P. Calafiura, A. Calandri, G. Calderini, P. Calfayan, L. P. Caloba, S. Calvente Lopez, D. Calvet, S. Calvet, T. P. Calvet, R. Camacho Toro, S. Camarda, P. Camarri, D. Cameron, R. Caminal Armadans, C. Camincher, S. Campana, M. Campanelli, A. Camplani, A. Campoverde, V. Canale, A. Canepa, M. Cano Bret, J. Cantero, R. Cantrill, T. Cao, M. D. M. Capeans Garrido, I. Caprini, M. Caprini, M. Capua, R. Caputo, R. M. Carbone, R. Cardarelli, F. Cardillo, I. Carli, T. Carli, G. Carlino, L. Carminati, S. Caron, E. Carquin, G. D. Carrillo-Montoya, J. R. Carter, J. Carvalho, D. Casadei, M. P. Casado, M. Casolino, D. W. Casper, E. Castaneda-Miranda, R. Castelijn, A. Castelli, V. Castillo Gimenez, N. F. Castro, A. Catinaccio, J. R. Catmore, A. Cattai, J. Caudron, V. Cavaliere, E. Cavallaro, D. Cavalli, M. Cavalli-Sforza, V. Cavasinni, F. Ceradini, L. Cerda Alberich, B. C. Cerio, A. S. Cerqueira, A. Cerri, L. Cerrito, F. Cerutti, M. Cerv, A. Cervelli, S. A. Cetin, A. Chafaq, D. Chakraborty, S. K. Chan, Y. L. Chan, P. Chang, J. D. Chapman, D. G. Charlton, A. Chatterjee, C. C. Chau, C. A. Chavez Barajas, S. Che, S. Cheatham, A. Chegwidden, S. Chekanov, S. V. Chekulaev, G. A. Chelkov, M. A. Chelstowska, C. Chen, H. Chen, K. Chen, S. Chen, S. Chen, X. Chen, Y. Chen, H. C. Cheng, H. J Cheng, Y. Cheng, A. Cheplakov, E. Cheremushkina, R. Cherkaoui El Moursli, V. Chernyatin, E. Cheu, L. Chevalier, V. Chiarella, G. Chiarelli, G. Chiodini, A. S. Chisholm, A. Chitan, M. V. Chizhov, K. Choi, A. R. Chomont, S. Chouridou, B. K. B. Chow, V. Christodoulou, D. Chromek-Burckhart, J. Chudoba, A. J. Chuinard, J. J. Chwastowski, L. Chytka, G. Ciapetti, A. K. Ciftci, D. Cinca, V. Cindro, I. A. Cioara, C. Ciocca, A. Ciocio, F. Cirotto, Z. H. Citron, M. Citterio, M. Ciubancan, A. Clark, B. L. Clark, M. R. Clark, P. J. Clark, R. N. Clarke, C. Clement, Y. Coadou, M. Cobal, A. Coccaro, J. Cochran, L. Coffey, L. Colasurdo, B. Cole, A. P. Colijn, J. Collot, T. Colombo, G. Compostella, P. Conde Muiño, E. Coniavitis, S. H. Connell, I. A. Connelly, V. Consorti, S. Constantinescu, G. Conti, F. Conventi, M. Cooke, B. D. Cooper, A. M. Cooper-Sarkar, K. J. R. Cormier, T. Cornelissen, M. Corradi, F. Corriveau, A. Corso-Radu, A. Cortes-Gonzalez, G. Cortiana, G. Costa, M. J. Costa, D. Costanzo, G. Cottin, G. Cowan, B. E. Cox, K. Cranmer, S. J. Crawley, G. Cree, S. Crépé-Renaudin, F. Crescioli, W. A. Cribbs, M. Crispin Ortuzar, M. Cristinziani, V. Croft, G. Crosetti, T. Cuhadar Donszelmann, J. Cummings, M. Curatolo, J. Cúth, C. Cuthbert, H. Czirr, P. Czodrowski, G. D’amen, S. D’Auria, M. D’Onofrio, M. J. Da Cunha Sargedas De Sousa, C. Da Via, W. Dabrowski, T. Dado, T. Dai, O. Dale, F. Dallaire, C. Dallapiccola, M. Dam, J. R. Dandoy, N. P. Dang, A. C. Daniells, N. S. Dann, M. Danninger, M. Dano Hoffmann, V. Dao, G. Darbo, S. Darmora, J. Dassoulas, A. Dattagupta, W. Davey, C. David, T. Davidek, M. Davies, P. Davison, E. Dawe, I. Dawson, R. K. Daya-Ishmukhametova, K. De, R. de Asmundis, A. De Benedetti, S. De Castro, S. De Cecco, N. De Groot, P. de Jong, H. De la Torre, F. De Lorenzi, A. De Maria, D. De Pedis, A. De Salvo, U. De Sanctis, A. De Santo, J. B. De Vivie De Regie, W. J. Dearnaley, R. Debbe, C. Debenedetti, D. V. Dedovich, N. Dehghanian, I. Deigaard, M. Del Gaudio, J. Del Peso, T. Del Prete, D. Delgove, F. Deliot, C. M. Delitzsch, M. Deliyergiyev, A. Dell’Acqua, L. Dell’Asta, M. Dell’Orso, M. Della Pietra, D. della Volpe, M. Delmastro, P. A. Delsart, D. A. DeMarco, S. Demers, M. Demichev, A. Demilly, S. P. Denisov, D. Denysiuk, D. Derendarz, J. E. Derkaoui, F. Derue, P. Dervan, K. Desch, C. Deterre, K. Dette, P. O. Deviveiros, A. Dewhurst, S. Dhaliwal, A. Di Ciaccio, L. Di Ciaccio, W. K. Di Clemente, C. Di Donato, A. Di Girolamo, B. Di Girolamo, B. Di Micco, R. Di Nardo, A. Di Simone, R. Di Sipio, D. Di Valentino, C. Diaconu, M. Diamond, F. A. Dias, M. A. Diaz, E. B. Diehl, J. Dietrich, S. Diglio, A. Dimitrievska, J. Dingfelder, P. Dita, S. Dita, F. Dittus, F. Djama, T. Djobava, J. I. Djuvsland, M. A. B. do Vale, D. Dobos, M. Dobre, C. Doglioni, T. Dohmae, J. Dolejsi, Z. Dolezal, B. A. Dolgoshein, M. Donadelli, S. Donati, P. Dondero, J. Donini, J. Dopke, A. Doria, M. T. Dova, A. T. Doyle, E. Drechsler, M. Dris, Y. Du, J. Duarte-Campderros, E. Duchovni, G. Duckeck, O. A. Ducu, D. Duda, A. Dudarev, E. M. Duffield, L. Duflot, L. Duguid, M. Dührssen, M. Dumancic, M. Dunford, H. Duran Yildiz, M. Düren, A. Durglishvili, D. Duschinger, B. Dutta, M. Dyndal, C. Eckardt, K. M. Ecker, R. C. Edgar, N. C. Edwards, T. Eifert, G. Eigen, K. Einsweiler, T. Ekelof, M. El Kacimi, V. Ellajosyula, M. Ellert, S. Elles, F. Ellinghaus, A. A. Elliot, N. Ellis, J. Elmsheuser, M. Elsing, D. Emeliyanov, Y. Enari, O. C. Endner, M. Endo, J. S. Ennis, J. Erdmann, A. Ereditato, G. Ernis, J. Ernst, M. Ernst, S. Errede, E. Ertel, M. Escalier, H. Esch, C. Escobar, B. Esposito, A. I. Etienvre, E. Etzion, H. Evans, A. Ezhilov, F. Fabbri, L. Fabbri, G. Facini, R. M. Fakhrutdinov, S. Falciano, R. J. Falla, J. Faltova, Y. Fang, M. Fanti, A. Farbin, A. Farilla, C. Farina, E. M. Farina, T. Farooque, S. Farrell, S. M. Farrington, P. Farthouat, F. Fassi, P. Fassnacht, D. Fassouliotis, M. Faucci Giannelli, A. Favareto, W. J. Fawcett, L. Fayard, O. L. Fedin, W. Fedorko, S. Feigl, L. Feligioni, C. Feng, E. J. Feng, H. Feng, A. B. Fenyuk, L. Feremenga, P. Fernandez Martinez, S. Fernandez Perez, J. Ferrando, A. Ferrari, P. Ferrari, R. Ferrari, D. E. Ferreira de Lima, A. Ferrer, D. Ferrere, C. Ferretti, A. Ferretto Parodi, F. Fiedler, A. Filipčič, M. Filipuzzi, F. Filthaut, M. Fincke-Keeler, K. D. Finelli, M. C. N. Fiolhais, L. Fiorini, A. Firan, A. Fischer, C. Fischer, J. Fischer, W. C. Fisher, N. Flaschel, I. Fleck, P. Fleischmann, G. T. Fletcher, R. R. M. Fletcher, T. Flick, A. Floderus, L. R. Flores Castillo, M. J. Flowerdew, G. T. Forcolin, A. Formica, A. Forti, A. G. Foster, D. Fournier, H. Fox, S. Fracchia, P. Francavilla, M. Franchini, D. Francis, L. Franconi, M. Franklin, M. Frate, M. Fraternali, D. Freeborn, S. M. Fressard-Batraneanu, F. Friedrich, D. Froidevaux, J. A. Frost, C. Fukunaga, E. Fullana Torregrosa, T. Fusayasu, J. Fuster, C. Gabaldon, O. Gabizon, A. Gabrielli, A. Gabrielli, G. P. Gach, S. Gadatsch, S. Gadomski, G. Gagliardi, L. G. Gagnon, P. Gagnon, C. Galea, B. Galhardo, E. J. Gallas, B. J. Gallop, P. Gallus, G. Galster, K. K. Gan, J. Gao, Y. Gao, Y. S. Gao, F. M. Garay Walls, C. García, J. E. García Navarro, M. Garcia-Sciveres, R. W. Gardner, N. Garelli, V. Garonne, A. Gascon Bravo, C. Gatti, A. Gaudiello, G. Gaudio, B. Gaur, L. Gauthier, I. L. Gavrilenko, C. Gay, G. Gaycken, E. N. Gazis, Z. Gecse, C. N. P. Gee, Ch. Geich-Gimbel, M. Geisen, M. P. Geisler, C. Gemme, M. H. Genest, C. Geng, S. Gentile, S. George, D. Gerbaudo, A. Gershon, S. Ghasemi, H. Ghazlane, M. Ghneimat, B. Giacobbe, S. Giagu, P. Giannetti, B. Gibbard, S. M. Gibson, M. Gignac, M. Gilchriese, T. P. S. Gillam, D. Gillberg, G. Gilles, D. M. Gingrich, N. Giokaris, M. P. Giordani, F. M. Giorgi, F. M. Giorgi, P. F. Giraud, P. Giromini, D. Giugni, F. Giuli, C. Giuliani, M. Giulini, B. K. Gjelsten, S. Gkaitatzis, I. Gkialas, E. L. Gkougkousis, L. K. Gladilin, C. Glasman, J. Glatzer, P. C. F. Glaysher, A. Glazov, M. Goblirsch-Kolb, J. Godlewski, S. Goldfarb, T. Golling, D. Golubkov, A. Gomes, R. Gonçalo, J. Goncalves Pinto Firmino Da Costa, G. Gonella, L. Gonella, A. Gongadze, S. González de la Hoz, G. Gonzalez Parra, S. Gonzalez-Sevilla, L. Goossens, P. A. Gorbounov, H. A. Gordon, I. Gorelov, B. Gorini, E. Gorini, A. Gorišek, E. Gornicki, A. T. Goshaw, C. Gössling, M. I. Gostkin, C. R. Goudet, D. Goujdami, A. G. Goussiou, N. Govender, E. Gozani, L. Graber, I. Grabowska-Bold, P. O. J. Gradin, P. Grafström, J. Gramling, E. Gramstad, S. Grancagnolo, V. Gratchev, P. M. Gravila, H. M. Gray, E. Graziani, Z. D. Greenwood, C. Grefe, K. Gregersen, I. M. Gregor, P. Grenier, K. Grevtsov, J. Griffiths, A. A. Grillo, K. Grimm, S. Grinstein, Ph. Gris, J.-F. Grivaz, S. Groh, J. P. Grohs, E. Gross, J. Grosse-Knetter, G. C. Grossi, Z. J. Grout, L. Guan, W. Guan, J. Guenther, F. Guescini, D. Guest, O. Gueta, E. Guido, T. Guillemin, S. Guindon, U. Gul, C. Gumpert, J. Guo, Y. Guo, S. Gupta, G. Gustavino, P. Gutierrez, N. G. Gutierrez Ortiz, C. Gutschow, C. Guyot, C. Gwenlan, C. B. Gwilliam, A. Haas, C. Haber, H. K. Hadavand, N. Haddad, A. Hadef, P. Haefner, S. Hageböck, Z. Hajduk, H. Hakobyan, M. Haleem, J. Haley, G. Halladjian, G. D. Hallewell, K. Hamacher, P. Hamal, K. Hamano, A. Hamilton, G. N. Hamity, P. G. Hamnett, L. Han, K. Hanagaki, K. Hanawa, M. Hance, B. Haney, P. Hanke, R. Hanna, J. B. Hansen, J. D. Hansen, M. C. Hansen, P. H. Hansen, K. Hara, A. S. Hard, T. Harenberg, F. Hariri, S. Harkusha, R. D. Harrington, P. F. Harrison, F. Hartjes, N. M. Hartmann, M. Hasegawa, Y. Hasegawa, A. Hasib, S. Hassani, S. Haug, R. Hauser, L. Hauswald, M. Havranek, C. M. Hawkes, R. J. Hawkings, D. Hayden, C. P. Hays, J. M. Hays, H. S. Hayward, S. J. Haywood, S. J. Head, T. Heck, V. Hedberg, L. Heelan, S. Heim, T. Heim, B. Heinemann, J. J. Heinrich, L. Heinrich, C. Heinz, J. Hejbal, L. Helary, S. Hellman, C. Helsens, J. Henderson, R. C. W. Henderson, Y. Heng, S. Henkelmann, A. M. Henriques Correia, S. Henrot-Versille, G. H. Herbert, Y. Hernández Jiménez, G. Herten, R. Hertenberger, L. Hervas, G. G. Hesketh, N. P. Hessey, J. W. Hetherly, R. Hickling, E. Higón-Rodriguez, E. Hill, J. C. Hill, K. H. Hiller, S. J. Hillier, I. Hinchliffe, E. Hines, R. R. Hinman, M. Hirose, D. Hirschbuehl, J. Hobbs, N. Hod, M. C. Hodgkinson, P. Hodgson, A. Hoecker, M. R. Hoeferkamp, F. Hoenig, D. Hohn, T. R. Holmes, M. Homann, T. M. Hong, B. H. Hooberman, W. H. Hopkins, Y. Horii, A. J. Horton, J-Y. Hostachy, S. Hou, A. Hoummada, J. Howarth, M. Hrabovsky, I. Hristova, J. Hrivnac, T. Hryn’ova, A. Hrynevich, C. Hsu, P. J. Hsu, S.-C. Hsu, D. Hu, Q. Hu, Y. Huang, Z. Hubacek, F. Hubaut, F. Huegging, T. B. Huffman, E. W. Hughes, G. Hughes, M. Huhtinen, P. Huo, N. Huseynov, J. Huston, J. Huth, G. Iacobucci, G. Iakovidis, I. Ibragimov, L. Iconomidou-Fayard, E. Ideal, Z. Idrissi, P. Iengo, O. Igonkina, T. Iizawa, Y. Ikegami, M. Ikeno, Y. Ilchenko, D. Iliadis, N. Ilic, T. Ince, G. Introzzi, P. Ioannou, M. Iodice, K. Iordanidou, V. Ippolito, N. Ishijima, M. Ishino, M. Ishitsuka, R. Ishmukhametov, C. Issever, S. Istin, F. Ito, J. M. Iturbe Ponce, R. Iuppa, W. Iwanski, H. Iwasaki, J. M. Izen, V. Izzo, S. Jabbar, B. Jackson, M. Jackson, P. Jackson, V. Jain, K. B. Jakobi, K. Jakobs, S. Jakobsen, T. Jakoubek, D. O. Jamin, D. K. Jana, E. Jansen, R. Jansky, J. Janssen, M. Janus, G. Jarlskog, N. Javadov, T. Javůrek, F. Jeanneau, L. Jeanty, G.-Y. Jeng, D. Jennens, P. Jenni, J. Jentzsch, C. Jeske, S. Jézéquel, H. Ji, J. Jia, H. Jiang, Y. Jiang, S. Jiggins, J. Jimenez Pena, S. Jin, A. Jinaru, O. Jinnouchi, P. Johansson, K. A. Johns, W. J. Johnson, K. Jon-And, G. Jones, R. W. L. Jones, S. Jones, T. J. Jones, J. Jongmanns, P. M. Jorge, J. Jovicevic, X. Ju, A. Juste Rozas, M. K. Köhler, A. Kaczmarska, M. Kado, H. Kagan, M. Kagan, S. J. Kahn, E. Kajomovitz, C. W. Kalderon, A. Kaluza, S. Kama, A. Kamenshchikov, N. Kanaya, S. Kaneti, L. Kanjir, V. A. Kantserov, J. Kanzaki, B. Kaplan, L. S. Kaplan, A. Kapliy, D. Kar, K. Karakostas, A. Karamaoun, N. Karastathis, M. J. Kareem, E. Karentzos, M. Karnevskiy, S. N. Karpov, Z. M. Karpova, K. Karthik, V. Kartvelishvili, A. N. Karyukhin, K. Kasahara, L. Kashif, R. D. Kass, A. Kastanas, Y. Kataoka, C. Kato, A. Katre, J. Katzy, K Kawade, K. Kawagoe, T. Kawamoto, G. Kawamura, S. Kazama, V. F. Kazanin, R. Keeler, R. Kehoe, J. S. Keller, J. J. Kempster, H. Keoshkerian, O. Kepka, B. P. Kerševan, S. Kersten, R. A. Keyes, M. Khader, F. Khalil-zada, A. Khanov, A. G. Kharlamov, T. J. Khoo, V. Khovanskiy, E. Khramov, J. Khubua, S. Kido, H. Y. Kim, S. H. Kim, Y. K. Kim, N. Kimura, O. M. Kind, B. T. King, M. King, S. B. King, J. Kirk, A. E. Kiryunin, T. Kishimoto, D. Kisielewska, F. Kiss, K. Kiuchi, O. Kivernyk, E. Kladiva, M. H. Klein, M. Klein, U. Klein, K. Kleinknecht, P. Klimek, A. Klimentov, R. Klingenberg, J. A. Klinger, T. Klioutchnikova, E.-E. Kluge, P. Kluit, S. Kluth, J. Knapik, E. Kneringer, E. B. F. G. Knoops, A. Knue, A. Kobayashi, D. Kobayashi, T. Kobayashi, M. Kobel, M. Kocian, P. Kodys, T. Koffas, E. Koffeman, T. Koi, H. Kolanoski, M. Kolb, I. Koletsou, A. A. Komar, Y. Komori, T. Kondo, N. Kondrashova, K. Köneke, A. C. König, T. Kono, R. Konoplich, N. Konstantinidis, R. Kopeliansky, S. Koperny, L. Köpke, A. K. Kopp, K. Korcyl, K. Kordas, A. Korn, A. A. Korol, I. Korolkov, E. V. Korolkova, O. Kortner, S. Kortner, T. Kosek, V. V. Kostyukhin, A. Kotwal, A. Kourkoumeli-Charalampidi, C. Kourkoumelis, V. Kouskoura, A. B. Kowalewska, R. Kowalewski, T. Z. Kowalski, C. Kozakai, W. Kozanecki, A. S. Kozhin, V. A. Kramarenko, G. Kramberger, D. Krasnopevtsev, M. W. Krasny, A. Krasznahorkay, J. K. Kraus, A. Kravchenko, M. Kretz, J. Kretzschmar, K. Kreutzfeldt, P. Krieger, K. Krizka, K. Kroeninger, H. Kroha, J. Kroll, J. Kroseberg, J. Krstic, U. Kruchonak, H. Krüger, N. Krumnack, A. Kruse, M. C. Kruse, M. Kruskal, T. Kubota, H. Kucuk, S. Kuday, J. T. Kuechler, S. Kuehn, A. Kugel, F. Kuger, A. Kuhl, T. Kuhl, V. Kukhtin, R. Kukla, Y. Kulchitsky, S. Kuleshov, M. Kuna, T. Kunigo, A. Kupco, H. Kurashige, Y. A. Kurochkin, V. Kus, E. S. Kuwertz, M. Kuze, J. Kvita, T. Kwan, D. Kyriazopoulos, A. La Rosa, J. L. La Rosa Navarro, L. La Rotonda, C. Lacasta, F. Lacava, J. Lacey, H. Lacker, D. Lacour, V. R. Lacuesta, E. Ladygin, R. Lafaye, B. Laforge, T. Lagouri, S. Lai, S. Lammers, W. Lampl, E. Lançon, U. Landgraf, M. P. J. Landon, V. S. Lang, J. C. Lange, A. J. Lankford, F. Lanni, K. Lantzsch, A. Lanza, S. Laplace, C. Lapoire, J. F. Laporte, T. Lari, F. Lasagni Manghi, M. Lassnig, P. Laurelli, W. Lavrijsen, A. T. Law, P. Laycock, T. Lazovich, M. Lazzaroni, B. Le, O. Le Dortz, E. Le Guirriec, E. P. Le Quilleuc, M. LeBlanc, T. LeCompte, F. Ledroit-Guillon, C. A. Lee, S. C. Lee, L. Lee, G. Lefebvre, M. Lefebvre, F. Legger, C. Leggett, A. Lehan, G. Lehmann Miotto, X. Lei, W. A. Leight, A. Leisos, A. G. Leister, M. A. L. Leite, R. Leitner, D. Lellouch, B. Lemmer, K. J. C. Leney, T. Lenz, B. Lenzi, R. Leone, S. Leone, C. Leonidopoulos, S. Leontsinis, G. Lerner, C. Leroy, A. A. J. Lesage, C. G. Lester, M. Levchenko, J. Levêque, D. Levin, L. J. Levinson, M. Levy, D. Lewis, A. M. Leyko, M. Leyton, B. Li, H. Li, H. L. Li, L. Li, L. Li, Q. Li, S. Li, X. Li, Y. Li, Z. Liang, B. Liberti, A. Liblong, P. Lichard, K. Lie, J. Liebal, W. Liebig, A. Limosani, S. C. Lin, T. H. Lin, B. E. Lindquist, A. E. Lionti, E. Lipeles, A. Lipniacka, M. Lisovyi, T. M. Liss, A. Lister, A. M. Litke, B. Liu, D. Liu, H. Liu, H. Liu, J. Liu, J. B. Liu, K. Liu, L. Liu, M. Liu, M. Liu, Y. L. Liu, Y. Liu, M. Livan, A. Lleres, J. Llorente Merino, S. L. Lloyd, F. Lo Sterzo, E. M. Lobodzinska, P. Loch, W. S. Lockman, F. K. Loebinger, A. E. Loevschall-Jensen, K. M. Loew, A. Loginov, T. Lohse, K. Lohwasser, M. Lokajicek, B. A. Long, J. D. Long, R. E. Long, L. Longo, K. A. Looper, L. Lopes, D. Lopez Mateos, B. Lopez Paredes, I. Lopez Paz, A. Lopez Solis, J. Lorenz, N. Lorenzo Martinez, M. Losada, P. J. Lösel, X. Lou, A. Lounis, J. Love, P. A. Love, H. Lu, N. Lu, H. J. Lubatti, C. Luci, A. Lucotte, C. Luedtke, F. Luehring, W. Lukas, L. Luminari, O. Lundberg, B. Lund-Jensen, P. M. Luzi, D. Lynn, R. Lysak, E. Lytken, V. Lyubushkin, H. Ma, L. L. Ma, Y. Ma, G. Maccarrone, A. Macchiolo, C. M. Macdonald, B. Maček, J. Machado Miguens, D. Madaffari, R. Madar, H. J. Maddocks, W. F. Mader, A. Madsen, J. Maeda, S. Maeland, T. Maeno, A. Maevskiy, E. Magradze, J. Mahlstedt, C. Maiani, C. Maidantchik, A. A. Maier, T. Maier, A. Maio, S. Majewski, Y. Makida, N. Makovec, B. Malaescu, Pa. Malecki, V. P. Maleev, F. Malek, U. Mallik, D. Malon, C. Malone, S. Maltezos, S. Malyukov, J. Mamuzic, G. Mancini, B. Mandelli, L. Mandelli, I. Mandić, J. Maneira, L. Manhaes de Andrade Filho, J. Manjarres Ramos, A. Mann, A. Manousos, B. Mansoulie, J. D. Mansour, R. Mantifel, M. Mantoani, S. Manzoni, L. Mapelli, G. Marceca, L. March, G. Marchiori, M. Marcisovsky, M. Marjanovic, D. E. Marley, F. Marroquim, S. P. Marsden, Z. Marshall, S. Marti-Garcia, B. Martin, T. A. Martin, V. J. Martin, B. Martin dit Latour, M. Martinez, V. I. Martinez Outschoorn, S. Martin-Haugh, V. S. Martoiu, A. C. Martyniuk, M. Marx, A. Marzin, L. Masetti, T. Mashimo, R. Mashinistov, J. Masik, A. L. Maslennikov, I. Massa, L. Massa, P. Mastrandrea, A. Mastroberardino, T. Masubuchi, P. Mättig, J. Mattmann, J. Maurer, S. J. Maxfield, D. A. Maximov, R. Mazini, S. M. Mazza, N. C. Mc Fadden, G. Mc Goldrick, S. P. Mc Kee, A. McCarn, R. L. McCarthy, T. G. McCarthy, L. I. McClymont, E. F. McDonald, K. W. McFarlane, J. A. Mcfayden, G. Mchedlidze, S. J. McMahon, R. A. McPherson, M. Medinnis, S. Meehan, S. Mehlhase, A. Mehta, K. Meier, C. Meineck, B. Meirose, D. Melini, B. R. Mellado Garcia, M. Melo, F. Meloni, A. Mengarelli, S. Menke, E. Meoni, S. Mergelmeyer, P. Mermod, L. Merola, C. Meroni, F. S. Merritt, A. Messina, J. Metcalfe, A. S. Mete, C. Meyer, C. Meyer, J-P. Meyer, J. Meyer, H. Meyer Zu Theenhausen, F. Miano, R. P. Middleton, S. Miglioranzi, L. Mijović, G. Mikenberg, M. Mikestikova, M. Mikuž, M. Milesi, A. Milic, D. W. Miller, C. Mills, A. Milov, D. A. Milstead, A. A. Minaenko, Y. Minami, I. A. Minashvili, A. I. Mincer, B. Mindur, M. Mineev, Y. Ming, L. M. Mir, K. P. Mistry, T. Mitani, J. Mitrevski, V. A. Mitsou, A. Miucci, P. S. Miyagawa, J. U. Mjörnmark, T. Moa, K. Mochizuki, S. Mohapatra, S. Molander, R. Moles-Valls, R. Monden, M. C. Mondragon, K. Mönig, J. Monk, E. Monnier, A. Montalbano, J. Montejo Berlingen, F. Monticelli, S. Monzani, R. W. Moore, N. Morange, D. Moreno, M. Moreno Llácer, P. Morettini, S. Morgenstern, D. Mori, T. Mori, M. Morii, M. Morinaga, V. Morisbak, S. Moritz, A. K. Morley, G. Mornacchi, J. D. Morris, S. S. Mortensen, L. Morvaj, M. Mosidze, J. Moss, K. Motohashi, R. Mount, E. Mountricha, S. V. Mouraviev, E. J. W. Moyse, S. Muanza, R. D. Mudd, F. Mueller, J. Mueller, R. S. P. Mueller, T. Mueller, D. Muenstermann, P. Mullen, G. A. Mullier, F. J. Munoz Sanchez, J. A. Murillo Quijada, W. J. Murray, H. Musheghyan, M. Muškinja, A. G. Myagkov, M. Myska, B. P. Nachman, O. Nackenhorst, K. Nagai, R. Nagai, K. Nagano, Y. Nagasaka, K. Nagata, M. Nagel, E. Nagy, A. M. Nairz, Y. Nakahama, K. Nakamura, T. Nakamura, I. Nakano, H. Namasivayam, R. F. Naranjo Garcia, R. Narayan, D. I. Narrias Villar, I. Naryshkin, T. Naumann, G. Navarro, R. Nayyar, H. A. Neal, P. Yu. Nechaeva, T. J. Neep, P. D. Nef, A. Negri, M. Negrini, S. Nektarijevic, C. Nellist, A. Nelson, S. Nemecek, P. Nemethy, A. A. Nepomuceno, M. Nessi, M. S. Neubauer, M. Neumann, R. M. Neves, P. Nevski, P. R. Newman, D. H. Nguyen, T. Nguyen Manh, R. B. Nickerson, R. Nicolaidou, J. Nielsen, A. Nikiforov, V. Nikolaenko, I. Nikolic-Audit, K. Nikolopoulos, J. K. Nilsen, P. Nilsson, Y. Ninomiya, A. Nisati, R. Nisius, T. Nobe, L. Nodulman, M. Nomachi, I. Nomidis, T. Nooney, S. Norberg, M. Nordberg, N. Norjoharuddeen, O. Novgorodova, S. Nowak, M. Nozaki, L. Nozka, K. Ntekas, E. Nurse, F. Nuti, F. O’grady, D. C. O’Neil, A. A. O’Rourke, V. O’Shea, F. G. Oakham, H. Oberlack, T. Obermann, J. Ocariz, A. Ochi, I. Ochoa, J. P. Ochoa-Ricoux, S. Oda, S. Odaka, H. Ogren, A. Oh, S. H. Oh, C. C. Ohm, H. Ohman, H. Oide, H. Okawa, Y. Okumura, T. Okuyama, A. Olariu, L. F. Oleiro Seabra, S. A. Olivares Pino, D. Oliveira Damazio, A. Olszewski, J. Olszowska, A. Onofre, K. Onogi, P. U. E. Onyisi, M. J. Oreglia, Y. Oren, D. Orestano, N. Orlando, R. S. Orr, B. Osculati, R. Ospanov, G. Otero y Garzon, H. Otono, M. Ouchrif, F. Ould-Saada, A. Ouraou, K. P. Oussoren, Q. Ouyang, M. Owen, R. E. Owen, V. E. Ozcan, N. Ozturk, K. Pachal, A. Pacheco Pages, L. Pacheco Rodriguez, C. Padilla Aranda, M. Pagáčová, S. Pagan Griso, F. Paige, P. Pais, K. Pajchel, G. Palacino, S. Palazzo, S. Palestini, M. Palka, D. Pallin, A. Palma, E. St. Panagiotopoulou, C. E. Pandini, J. G. Panduro Vazquez, P. Pani, S. Panitkin, D. Pantea, L. Paolozzi, Th. D. Papadopoulou, K. Papageorgiou, A. Paramonov, D. Paredes Hernandez, A. J. Parker, M. A. Parker, K. A. Parker, F. Parodi, J. A. Parsons, U. Parzefall, V. R. Pascuzzi, E. Pasqualucci, S. Passaggio, Fr. Pastore, G. Pásztor, S. Pataraia, J. R. Pater, T. Pauly, J. Pearce, B. Pearson, L. E. Pedersen, M. Pedersen, S. Pedraza Lopez, R. Pedro, S. V. Peleganchuk, D. Pelikan, O. Penc, C. Peng, H. Peng, J. Penwell, B. S. Peralva, M. M. Perego, D. V. Perepelitsa, E. Perez Codina, L. Perini, H. Pernegger, S. Perrella, R. Peschke, V. D. Peshekhonov, K. Peters, R. F. Y. Peters, B. A. Petersen, T. C. Petersen, E. Petit, A. Petridis, C. Petridou, P. Petroff, E. Petrolo, M. Petrov, F. Petrucci, N. E. Pettersson, A. Peyaud, R. Pezoa, P. W. Phillips, G. Piacquadio, E. Pianori, A. Picazio, E. Piccaro, M. Piccinini, M. A. Pickering, R. Piegaia, J. E. Pilcher, A. D. Pilkington, A. W. J. Pin, M. Pinamonti, J. L. Pinfold, A. Pingel, S. Pires, H. Pirumov, M. Pitt, L. Plazak, M.-A. Pleier, V. Pleskot, E. Plotnikova, P. Plucinski, D. Pluth, R. Poettgen, L. Poggioli, D. Pohl, G. Polesello, A. Poley, A. Policicchio, R. Polifka, A. Polini, C. S. Pollard, V. Polychronakos, K. Pommès, L. Pontecorvo, B. G. Pope, G. A. Popeneciu, D. S. Popovic, A. Poppleton, S. Pospisil, K. Potamianos, I. N. Potrap, C. J. Potter, C. T. Potter, G. Poulard, J. Poveda, V. Pozdnyakov, M. E. Pozo Astigarraga, P. Pralavorio, A. Pranko, S. Prell, D. Price, L. E. Price, M. Primavera, S. Prince, M. Proissl, K. Prokofiev, F. Prokoshin, S. Protopopescu, J. Proudfoot, M. Przybycien, D. Puddu, M. Purohit, P. Puzo, J. Qian, G. Qin, Y. Qin, A. Quadt, W. B. Quayle, M. Queitsch-Maitland, D. Quilty, S. Raddum, V. Radeka, V. Radescu, S. K. Radhakrishnan, P. Radloff, P. Rados, F. Ragusa, G. Rahal, J. A. Raine, S. Rajagopalan, M. Rammensee, C. Rangel-Smith, M. G. Ratti, F. Rauscher, S. Rave, T. Ravenscroft, I. Ravinovich, M. Raymond, A. L. Read, N. P. Readioff, M. Reale, D. M. Rebuzzi, A. Redelbach, G. Redlinger, R. Reece, K. Reeves, L. Rehnisch, J. Reichert, H. Reisin, C. Rembser, H. Ren, M. Rescigno, S. Resconi, O. L. Rezanova, P. Reznicek, R. Rezvani, R. Richter, S. Richter, E. Richter-Was, O. Ricken, M. Ridel, P. Rieck, C. J. Riegel, J. Rieger, O. Rifki, M. Rijssenbeek, A. Rimoldi, M. Rimoldi, L. Rinaldi, B. Ristić, E. Ritsch, I. Riu, F. Rizatdinova, E. Rizvi, C. Rizzi, S. H. Robertson, A. Robichaud-Veronneau, D. Robinson, J. E. M. Robinson, A. Robson, C. Roda, Y. Rodina, A. Rodriguez Perez, D. Rodriguez Rodriguez, S. Roe, C. S. Rogan, O. Røhne, A. Romaniouk, M. Romano, S. M. Romano Saez, E. Romero Adam, N. Rompotis, M. Ronzani, L. Roos, E. Ros, S. Rosati, K. Rosbach, P. Rose, O. Rosenthal, N.-A. Rosien, V. Rossetti, E. Rossi, L. P. Rossi, J. H. N. Rosten, R. Rosten, M. Rotaru, I. Roth, J. Rothberg, D. Rousseau, C. R. Royon, A. Rozanov, Y. Rozen, X. Ruan, F. Rubbo, M. S. Rudolph, F. Rühr, A. Ruiz-Martinez, Z. Rurikova, N. A. Rusakovich, A. Ruschke, H. L. Russell, J. P. Rutherfoord, N. Ruthmann, Y. F. Ryabov, M. Rybar, G. Rybkin, S. Ryu, A. Ryzhov, G. F. Rzehorz, A. F. Saavedra, G. Sabato, S. Sacerdoti, H. F-W. Sadrozinski, R. Sadykov, F. Safai Tehrani, P. Saha, M. Sahinsoy, M. Saimpert, T. Saito, H. Sakamoto, Y. Sakurai, G. Salamanna, A. Salamon, J. E. Salazar Loyola, D. Salek, P. H. Sales De Bruin, D. Salihagic, A. Salnikov, J. Salt, D. Salvatore, F. Salvatore, A. Salvucci, A. Salzburger, D. Sammel, D. Sampsonidis, A. Sanchez, J. Sánchez, V. Sanchez Martinez, H. Sandaker, R. L. Sandbach, H. G. Sander, M. Sandhoff, C. Sandoval, R. Sandstroem, D. P. C. Sankey, M. Sannino, A. Sansoni, C. Santoni, R. Santonico, H. Santos, I. Santoyo Castillo, K. Sapp, A. Sapronov, J. G. Saraiva, B. Sarrazin, O. Sasaki, Y. Sasaki, K. Sato, G. Sauvage, E. Sauvan, G. Savage, P. Savard, C. Sawyer, L. Sawyer, J. Saxon, C. Sbarra, A. Sbrizzi, T. Scanlon, D. A. Scannicchio, M. Scarcella, V. Scarfone, J. Schaarschmidt, P. Schacht, B. M. Schachtner, D. Schaefer, R. Schaefer, J. Schaeffer, S. Schaepe, S. Schaetzel, U. Schäfer, A. C. Schaffer, D. Schaile, R. D. Schamberger, V. Scharf, V. A. Schegelsky, D. Scheirich, M. Schernau, C. Schiavi, S. Schier, C. Schillo, M. Schioppa, S. Schlenker, K. R. Schmidt-Sommerfeld, K. Schmieden, C. Schmitt, S. Schmitt, S. Schmitz, B. Schneider, U. Schnoor, L. Schoeffel, A. Schoening, B. D. Schoenrock, E. Schopf, M. Schott, J. Schovancova, S. Schramm, M. Schreyer, N. Schuh, A. Schulte, M. J. Schultens, H.-C. Schultz-Coulon, H. Schulz, M. Schumacher, B. A. Schumm, Ph. Schune, A. Schwartzman, T. A. Schwarz, Ph. Schwegler, H. Schweiger, Ph. Schwemling, R. Schwienhorst, J. Schwindling, T. Schwindt, G. Sciolla, F. Scuri, F. Scutti, J. Searcy, P. Seema, S. C. Seidel, A. Seiden, F. Seifert, J. M. Seixas, G. Sekhniaidze, K. Sekhon, S. J. Sekula, D. M. Seliverstov, N. Semprini-Cesari, C. Serfon, L. Serin, L. Serkin, M. Sessa, R. Seuster, H. Severini, T. Sfiligoj, F. Sforza, A. Sfyrla, E. Shabalina, N. W. Shaikh, L. Y. Shan, R. Shang, J. T. Shank, M. Shapiro, P. B. Shatalov, K. Shaw, S. M. Shaw, A. Shcherbakova, C. Y. Shehu, P. Sherwood, L. Shi, S. Shimizu, C. O. Shimmin, M. Shimojima, M. Shiyakova, A. Shmeleva, D. Shoaleh Saadi, M. J. Shochet, S. Shojaii, S. Shrestha, E. Shulga, M. A. Shupe, P. Sicho, A. M. Sickles, P. E. Sidebo, O. Sidiropoulou, D. Sidorov, A. Sidoti, F. Siegert, Dj. Sijacki, J. Silva, S. B. Silverstein, V. Simak, O. Simard, Lj. Simic, S. Simion, E. Simioni, B. Simmons, D. Simon, M. Simon, P. Sinervo, N. B. Sinev, M. Sioli, G. Siragusa, S. Yu. Sivoklokov, J. Sjölin, M. B. Skinner, H. P. Skottowe, P. Skubic, M. Slater, T. Slavicek, M. Slawinska, K. Sliwa, R. Slovak, V. Smakhtin, B. H. Smart, L. Smestad, J. Smiesko, S. Yu. Smirnov, Y. Smirnov, L. N. Smirnova, O. Smirnova, M. N. K. Smith, R. W. Smith, M. Smizanska, K. Smolek, A. A. Snesarev, S. Snyder, R. Sobie, F. Socher, A. Soffer, D. A. Soh, G. Sokhrannyi, C. A. Solans Sanchez, M. Solar, E. Yu. Soldatov, U. Soldevila, A. A. Solodkov, A. Soloshenko, O. V. Solovyanov, V. Solovyev, P. Sommer, H. Son, H. Y. Song, A. Sood, A. Sopczak, V. Sopko, V. Sorin, D. Sosa, C. L. Sotiropoulou, R. Soualah, A. M. Soukharev, D. South, B. C. Sowden, S. Spagnolo, M. Spalla, M. Spangenberg, F. Spanò, D. Sperlich, F. Spettel, R. Spighi, G. Spigo, L. A. Spiller, M. Spousta, R. D. St. Denis, A. Stabile, R. Stamen, S. Stamm, E. Stanecka, R. W. Stanek, C. Stanescu, M. Stanescu-Bellu, M. M. Stanitzki, S. Stapnes, E. A. Starchenko, G. H. Stark, J. Stark, P. Staroba, P. Starovoitov, S. Stärz, R. Staszewski, P. Steinberg, B. Stelzer, H. J. Stelzer, O. Stelzer-Chilton, H. Stenzel, G. A. Stewart, J. A. Stillings, M. C. Stockton, M. Stoebe, G. Stoicea, P. Stolte, S. Stonjek, A. R. Stradling, A. Straessner, M. E. Stramaglia, J. Strandberg, S. Strandberg, A. Strandlie, M. Strauss, P. Strizenec, R. Ströhmer, D. M. Strom, R. Stroynowski, A. Strubig, S. A. Stucci, B. Stugu, N. A. Styles, D. Su, J. Su, R. Subramaniam, S. Suchek, Y. Sugaya, M. Suk, V. V. Sulin, S. Sultansoy, T. Sumida, S. Sun, X. Sun, J. E. Sundermann, K. Suruliz, G. Susinno, M. R. Sutton, S. Suzuki, M. Svatos, M. Swiatlowski, I. Sykora, T. Sykora, D. Ta, C. Taccini, K. Tackmann, J. Taenzer, A. Taffard, R. Tafirout, N. Taiblum, H. Takai, R. Takashima, T. Takeshita, Y. Takubo, M. Talby, A. A. Talyshev, K. G. Tan, J. Tanaka, R. Tanaka, S. Tanaka, B. B. Tannenwald, S. Tapia Araya, S. Tapprogge, S. Tarem, G. F. Tartarelli, P. Tas, M. Tasevsky, T. Tashiro, E. Tassi, A. Tavares Delgado, Y. Tayalati, A. C. Taylor, G. N. Taylor, P. T. E. Taylor, W. Taylor, F. A. Teischinger, P. Teixeira-Dias, K. K. Temming, D. Temple, H. Ten Kate, P. K. Teng, J. J. Teoh, F. Tepel, S. Terada, K. Terashi, J. Terron, S. Terzo, M. Testa, R. J. Teuscher, T. Theveneaux-Pelzer, J. P. Thomas, J. Thomas-Wilsker, E. N. Thompson, P. D. Thompson, A. S. Thompson, L. A. Thomsen, E. Thomson, M. Thomson, M. J. Tibbetts, R. E. Ticse Torres, V. O. Tikhomirov, Yu. A. Tikhonov, S. Timoshenko, P. Tipton, S. Tisserant, K. Todome, T. Todorov, S. Todorova-Nova, J. Tojo, S. Tokár, K. Tokushuku, E. Tolley, L. Tomlinson, M. Tomoto, L. Tompkins, K. Toms, B. Tong, E. Torrence, H. Torres, E. Torró Pastor, J. Toth, F. Touchard, D. R. Tovey, T. Trefzger, A. Tricoli, I. M. Trigger, S. Trincaz-Duvoid, M. F. Tripiana, W. Trischuk, B. Trocmé, A. Trofymov, C. Troncon, M. Trottier-McDonald, M. Trovatelli, L. Truong, M. Trzebinski, A. Trzupek, J. C-L. Tseng, P. V. Tsiareshka, G. Tsipolitis, N. Tsirintanis, S. Tsiskaridze, V. Tsiskaridze, E. G. Tskhadadze, K. M. Tsui, I. I. Tsukerman, V. Tsulaia, S. Tsuno, D. Tsybychev, A. Tudorache, V. Tudorache, A. N. Tuna, S. A. Tupputi, S. Turchikhin, D. Turecek, D. Turgeman, R. Turra, A. J. Turvey, P. M. Tuts, M. Tyndel, G. Ucchielli, I. Ueda, M. Ughetto, F. Ukegawa, G. Unal, A. Undrus, G. Unel, F. C. Ungaro, Y. Unno, C. Unverdorben, J. Urban, P. Urquijo, P. Urrejola, G. Usai, A. Usanova, L. Vacavant, V. Vacek, B. Vachon, C. Valderanis, E. Valdes Santurio, N. Valencic, S. Valentinetti, A. Valero, L. Valery, S. Valkar, S. Vallecorsa, J. A. Valls Ferrer, W. Van Den Wollenberg, P. C. Van Der Deijl, R. van der Geer, H. van der Graaf, N. van Eldik, P. van Gemmeren, J. Van Nieuwkoop, I. van Vulpen, M. C. van Woerden, M. Vanadia, W. Vandelli, R. Vanguri, A. Vaniachine, P. Vankov, G. Vardanyan, R. Vari, E. W. Varnes, T. Varol, D. Varouchas, A. Vartapetian, K. E. Varvell, J. G. Vasquez, F. Vazeille, T. Vazquez Schroeder, J. Veatch, L. M. Veloce, F. Veloso, S. Veneziano, A. Ventura, M. Venturi, N. Venturi, A. Venturini, V. Vercesi, M. Verducci, W. Verkerke, J. C. Vermeulen, A. Vest, M. C. Vetterli, O. Viazlo, I. Vichou, T. Vickey, O. E. Vickey Boeriu, G. H. A. Viehhauser, S. Viel, L. Vigani, R. Vigne, M. Villa, M. Villaplana Perez, E. Vilucchi, M. G. Vincter, V. B. Vinogradov, C. Vittori, I. Vivarelli, S. Vlachos, M. Vlasak, M. Vogel, P. Vokac, G. Volpi, M. Volpi, H. von der Schmitt, E. von Toerne, V. Vorobel, K. Vorobev, M. Vos, R. Voss, J. H. Vossebeld, N. Vranjes, M. Vranjes Milosavljevic, V. Vrba, M. Vreeswijk, R. Vuillermet, I. Vukotic, Z. Vykydal, P. Wagner, W. Wagner, H. Wahlberg, S. Wahrmund, J. Wakabayashi, J. Walder, R. Walker, W. Walkowiak, V. Wallangen, C. Wang, C. Wang, F. Wang, H. Wang, H. Wang, J. Wang, J. Wang, K. Wang, R. Wang, S. M. Wang, T. Wang, T. Wang, W. Wang, X. Wang, C. Wanotayaroj, A. Warburton, C. P. Ward, D. R. Wardrope, A. Washbrook, P. M. Watkins, A. T. Watson, M. F. Watson, G. Watts, S. Watts, B. M. Waugh, S. Webb, M. S. Weber, S. W. Weber, J. S. Webster, A. R. Weidberg, B. Weinert, J. Weingarten, C. Weiser, H. Weits, P. S. Wells, T. Wenaus, T. Wengler, S. Wenig, N. Wermes, M. Werner, M. D. Werner, P. Werner, M. Wessels, J. Wetter, K. Whalen, N. L. Whallon, A. M. Wharton, A. White, M. J. White, R. White, D. Whiteson, F. J. Wickens, W. Wiedenmann, M. Wielers, P. Wienemann, C. Wiglesworth, L. A. M. Wiik-Fuchs, A. Wildauer, F. Wilk, H. G. Wilkens, H. H. Williams, S. Williams, C. Willis, S. Willocq, J. A. Wilson, I. Wingerter-Seez, F. Winklmeier, O. J. Winston, B. T. Winter, M. Wittgen, J. Wittkowski, M. W. Wolter, H. Wolters, S. D. Worm, B. K. Wosiek, J. Wotschack, M. J. Woudstra, K. W. Wozniak, M. Wu, M. Wu, S. L. Wu, X. Wu, Y. Wu, T. R. Wyatt, B. M. Wynne, S. Xella, D. Xu, L. Xu, B. Yabsley, S. Yacoob, R. Yakabe, D. Yamaguchi, Y. Yamaguchi, A. Yamamoto, S. Yamamoto, T. Yamanaka, K. Yamauchi, Y. Yamazaki, Z. Yan, H. Yang, H. Yang, Y. Yang, Z. Yang, W-M. Yao, Y. C. Yap, Y. Yasu, E. Yatsenko, K. H. Yau Wong, J. Ye, S. Ye, I. Yeletskikh, A. L. Yen, E. Yildirim, K. Yorita, R. Yoshida, K. Yoshihara, C. Young, C. J. S. Young, S. Youssef, D. R. Yu, J. Yu, J. M. Yu, J. Yu, L. Yuan, S. P. Y. Yuen, I. Yusuff, B. Zabinski, R. Zaidan, A. M. Zaitsev, N. Zakharchuk, J. Zalieckas, A. Zaman, S. Zambito, L. Zanello, D. Zanzi, C. Zeitnitz, M. Zeman, A. Zemla, J. C. Zeng, Q. Zeng, K. Zengel, O. Zenin, T. Ženiš, D. Zerwas, D. Zhang, F. Zhang, G. Zhang, H. Zhang, J. Zhang, L. Zhang, R. Zhang, R. Zhang, X. Zhang, Z. Zhang, X. Zhao, Y. Zhao, Z. Zhao, A. Zhemchugov, J. Zhong, B. Zhou, C. Zhou, L. Zhou, L. Zhou, M. Zhou, N. Zhou, C. G. Zhu, H. Zhu, J. Zhu, Y. Zhu, X. Zhuang, K. Zhukov, A. Zibell, D. Zieminska, N. I. Zimine, C. Zimmermann, S. Zimmermann, Z. Zinonos, M. Zinser, M. Ziolkowski, L. Živković, G. Zobernig, A. Zoccoli, M. zur Nedden, L. Zwalinski, ATLAS Collaboration

**Affiliations:** 1 Department of Physics, University of Adelaide, Adelaide, SA Australia; 2 Physics Department, SUNY Albany, Albany, NY USA; 3 Department of Physics, University of Alberta, Edmonton, AB Canada; 4 Department of Physics, Ankara University, Ankara, Turkey; 5 Istanbul Aydin University, Istanbul, Turkey; 6 Division of Physics, TOBB University of Economics and Technology, Ankara, Turkey; 7 LAPP, CNRS/IN2P3 and Université Savoie Mont Blanc, Annecy-le-Vieux, France; 8 High Energy Physics Division, Argonne National Laboratory, Argonne, IL USA; 9 Department of Physics, University of Arizona, Tucson, AZ USA; 10 Department of Physics, The University of Texas at Arlington, Arlington, TX USA; 11 Physics Department, University of Athens, Athens, Greece; 12 Physics Department, National Technical University of Athens, Zografou, Greece; 13 Department of Physics, The University of Texas at Austin, Austin, TX USA; 14 Institute of Physics, Azerbaijan Academy of Sciences, Baku, Azerbaijan; 15 Institut de Física d’Altes Energies (IFAE), The Barcelona Institute of Science and Technology, Barcelona, Spain; 16 Institute of Physics, University of Belgrade, Belgrade, Serbia; 17 Department for Physics and Technology, University of Bergen, Bergen, Norway; 18 Physics Division, Lawrence Berkeley National Laboratory and University of California, Berkeley, CA USA; 19 Department of Physics, Humboldt University, Berlin, Germany; 20 Albert Einstein Center for Fundamental Physics and Laboratory for High Energy Physics, University of Bern, Bern, Switzerland; 21 School of Physics and Astronomy, University of Birmingham, Birmingham, UK; 22 Department of Physics, Bogazici University, Istanbul, Turkey; 23 Department of Physics Engineering, Gaziantep University, Gaziantep, Turkey; 24 Istanbul Bilgi University, Faculty of Engineering and Natural Sciences, Istanbul, Turkey; 25 Bahcesehir University, Faculty of Engineering and Natural Sciences, Istanbul, Turkey; 26 Centro de Investigaciones, Universidad Antonio Narino, Bogota, Colombia; 27 INFN Sezione di Bologna, Bologna, Italy; 28 Dipartimento di Fisica e Astronomia, Università di Bologna, Bologna, Italy; 29 Physikalisches Institut, University of Bonn, Bonn, Germany; 30 Department of Physics, Boston University, Boston, MA USA; 31 Department of Physics, Brandeis University, Waltham, MA USA; 32 Universidade Federal do Rio De Janeiro COPPE/EE/IF, Rio de Janeiro, Brazil; 33 Electrical Circuits Department, Federal University of Juiz de Fora (UFJF), Juiz de Fora, Brazil; 34 Federal University of Sao Joao del Rei (UFSJ), Sao Joao del Rei, Brazil; 35 Instituto de Fisica, Universidade de Sao Paulo, Sao Paulo, Brazil; 36 Physics Department, Brookhaven National Laboratory, Upton, NY USA; 37 Transilvania University of Brasov, Brasov, Romania; 38 National Institute of Physics and Nuclear Engineering, Bucharest, Romania; 39 Physics Department, National Institute for Research and Development of Isotopic and Molecular Technologies, Cluj Napoca, Romania; 40 University Politehnica Bucharest, Bucharest, Romania; 41 West University in Timisoara, Timisoara, Romania; 42 Departamento de Física, Universidad de Buenos Aires, Buenos Aires, Argentina; 43 Cavendish Laboratory, University of Cambridge, Cambridge, UK; 44 Department of Physics, Carleton University, Ottawa, ON Canada; 45 CERN, Geneva, Switzerland; 46 Enrico Fermi Institute, University of Chicago, Chicago, IL USA; 47 Departamento de Física, Pontificia Universidad Católica de Chile, Santiago, Chile; 48 Departamento de Física, Universidad Técnica Federico Santa María, Valparaiso, Chile; 49 Institute of High Energy Physics, Chinese Academy of Sciences, Beijing, China; 50 Department of Physics, Nanjing University, Jiangsu, China; 51 Physics Department, Tsinghua University, Beijing, 100084 China; 52 Laboratoire de Physique Corpusculaire, Clermont Université and Université Blaise Pascal and CNRS/IN2P3, Clermont-Ferrand, France; 53 Nevis Laboratory, Columbia University, Irvington, NY USA; 54 Niels Bohr Institute, University of Copenhagen, Copenhagen, Denmark; 55 INFN Gruppo Collegato di Cosenza, Laboratori Nazionali di Frascati, Frascati, Italy; 56 Dipartimento di Fisica, Università della Calabria, Rende, Italy; 57 Faculty of Physics and Applied Computer Science, AGH University of Science and Technology, Krakow, Poland; 58 Marian Smoluchowski Institute of Physics, Jagiellonian University, Krakow, Poland; 59 Institute of Nuclear Physics, Polish Academy of Sciences, Krakow, Poland; 60 Physics Department, Southern Methodist University, Dallas, TX USA; 61 Physics Department, University of Texas at Dallas, Richardson, TX USA; 62 DESY, Hamburg and Zeuthen, Germany; 63 Lehrstuhl für Experimentelle Physik IV, Technische Universität Dortmund, Dortmund, Germany; 64 Institut für Kern-und Teilchenphysik, Technische Universität Dresden, Dresden, Germany; 65 Department of Physics, Duke University, Durham, NC USA; 66 SUPA-School of Physics and Astronomy, University of Edinburgh, Edinburgh, UK; 67 INFN Laboratori Nazionali di Frascati, Frascati, Italy; 68 Fakultät für Mathematik und Physik, Albert-Ludwigs-Universität, Freiburg, Germany; 69 Section de Physique, Université de Genève, Geneva, Switzerland; 70 INFN Sezione di Genova, Genoa, Italy; 71 Dipartimento di Fisica, Università di Genova, Genoa, Italy; 72 E. Andronikashvili Institute of Physics, Iv. Javakhishvili Tbilisi State University, Tbilisi, Georgia; 73 High Energy Physics Institute, Tbilisi State University, Tbilisi, Georgia; 74 II Physikalisches Institut, Justus-Liebig-Universität Giessen, Giessen, Germany; 75 SUPA-School of Physics and Astronomy, University of Glasgow, Glasgow, UK; 76 II Physikalisches Institut, Georg-August-Universität, Göttingen, Germany; 77 Laboratoire de Physique Subatomique et de Cosmologie, Université Grenoble-Alpes, CNRS/IN2P3, Grenoble, France; 78 Department of Physics, Hampton University, Hampton, VA USA; 79 Laboratory for Particle Physics and Cosmology, Harvard University, Cambridge, MA USA; 80 Department of Modern Physics, University of Science and Technology of China, Anhui, China; 81 Kirchhoff-Institut für Physik, Ruprecht-Karls-Universität Heidelberg, Heidelberg, Germany; 82 Physikalisches Institut, Ruprecht-Karls-Universität Heidelberg, Heidelberg, Germany; 83 ZITI Institut für technische Informatik, Ruprecht-Karls-Universität Heidelberg, Mannheim, Germany; 84 Faculty of Applied Information Science, Hiroshima Institute of Technology, Hiroshima, Japan; 85 Department of Physics, The Chinese University of Hong Kong, Shatin, NT Hong Kong; 86 Department of Physics, The University of Hong Kong, Hong Kong, China; 87 Department of Physics, The Hong Kong University of Science and Technology, Clear Water Bay, Kowloon, Hong Kong, China; 88 Department of Physics, Indiana University, Bloomington, IN USA; 89 Institut für Astro- und Teilchenphysik, Leopold-Franzens-Universität, Innsbruck, Austria; 90 University of Iowa, Iowa City, IA USA; 91 Department of Physics and Astronomy, Iowa State University, Ames, IA USA; 92 Joint Institute for Nuclear Research, JINR Dubna, Dubna, Russia; 93 KEK, High Energy Accelerator Research Organization, Tsukuba, Japan; 94 Graduate School of Science, Kobe University, Kobe, Japan; 95 Faculty of Science, Kyoto University, Kyoto, Japan; 96 Kyoto University of Education, Kyoto, Japan; 97 Department of Physics, Kyushu University, Fukuoka, Japan; 98 Instituto de Física La Plata, Universidad Nacional de La Plata and CONICET, La Plata, Argentina; 99 Physics Department, Lancaster University, Lancaster, UK; 100 INFN Sezione di Lecce, Lecce, Italy; 101 Dipartimento di Matematica e Fisica, Università del Salento, Lecce, Italy; 102 Oliver Lodge Laboratory, University of Liverpool, Liverpool, UK; 103 Department of Physics, Jožef Stefan Institute and University of Ljubljana, Ljubljana, Slovenia; 104 School of Physics and Astronomy, Queen Mary University of London, London, UK; 105 Department of Physics, Royal Holloway University of London, Surrey, UK; 106 Department of Physics and Astronomy, University College London, London, UK; 107 Louisiana Tech University, Ruston, LA USA; 108 Laboratoire de Physique Nucléaire et de Hautes Energies, UPMC and Université Paris-Diderot and CNRS/IN2P3, Paris, France; 109 Fysiska institutionen, Lunds universitet, Lund, Sweden; 110 Departamento de Fisica Teorica C-15, Universidad Autonoma de Madrid, Madrid, Spain; 111 Institut für Physik, Universität Mainz, Mainz, Germany; 112 School of Physics and Astronomy, University of Manchester, Manchester, UK; 113 CPPM, Aix-Marseille Université and CNRS/IN2P3, Marseille, France; 114 Department of Physics, University of Massachusetts, Amherst, MA USA; 115 Department of Physics, McGill University, Montreal, QC Canada; 116 School of Physics, University of Melbourne, Melbourne, VIC Australia; 117 Department of Physics, The University of Michigan, Ann Arbor, MI USA; 118 Department of Physics and Astronomy, Michigan State University, East Lansing, MI USA; 119 INFN Sezione di Milano, Milan, Italy; 120 Dipartimento di Fisica, Università di Milano, Milan, Italy; 121 B.I. Stepanov Institute of Physics, National Academy of Sciences of Belarus, Minsk, Republic of Belarus; 122 National Scientific and Educational Centre for Particle and High Energy Physics, Minsk, Republic of Belarus; 123 Group of Particle Physics, University of Montreal, Montreal, QC Canada; 124 P.N. Lebedev Physical Institute of the Russian Academy of Sciences, Moscow, Russia; 125 Institute for Theoretical and Experimental Physics (ITEP), Moscow, Russia; 126 National Research Nuclear University MEPhI, Moscow, Russia; 127 D.V. Skobeltsyn Institute of Nuclear Physics, M.V. Lomonosov Moscow State University, Moscow, Russia; 128 Fakultät für Physik, Ludwig-Maximilians-Universität München, München, Germany; 129 Max-Planck-Institut für Physik (Werner-Heisenberg-Institut), München, Germany; 130 Nagasaki Institute of Applied Science, Nagasaki, Japan; 131 Graduate School of Science and Kobayashi-Maskawa Institute, Nagoya University, Nagoya, Japan; 132 INFN Sezione di Napoli, Naples, Italy; 133 Dipartimento di Fisica, Università di Napoli, Naples, Italy; 134 Department of Physics and Astronomy, University of New Mexico, Albuquerque, NM USA; 135 Institute for Mathematics, Astrophysics and Particle Physics, Radboud University Nijmegen/Nikhef, Nijmegen, The Netherlands; 136 Nikhef National Institute for Subatomic Physics and University of Amsterdam, Amsterdam, The Netherlands; 137 Department of Physics, Northern Illinois University, DeKalb, IL USA; 138 Budker Institute of Nuclear Physics, SB RAS, Novosibirsk, Russia; 139 Department of Physics, New York University, New York, NY USA; 140 Ohio State University, Columbus, OH USA; 141 Faculty of Science, Okayama University, Okayama, Japan; 142 Homer L. Dodge Department of Physics and Astronomy, University of Oklahoma, Norman, OK USA; 143 Department of Physics, Oklahoma State University, Stillwater, OK USA; 144 Palacký University, RCPTM, Olomouc, Czech Republic; 145 Center for High Energy Physics, University of Oregon, Eugene, OR USA; 146 LAL, Univ. Paris-Sud, CNRS/IN2P3, Université Paris-Saclay, Orsay, France; 147 Graduate School of Science, Osaka University, Osaka, Japan; 148 Department of Physics, University of Oslo, Oslo, Norway; 149 Department of Physics, Oxford University, Oxford, UK; 150 INFN Sezione di Pavia, Pavia, Italy; 151 Dipartimento di Fisica, Università di Pavia, Pavia, Italy; 152 Department of Physics, University of Pennsylvania, Philadelphia, PA USA; 153 National Research Centre “Kurchatov Institute” B.P.Konstantinov Petersburg Nuclear Physics Institute, St. Petersburg, Russia; 154 INFN Sezione di Pisa, Pisa, Italy; 155 Dipartimento di Fisica E. Fermi, Università di Pisa, Pisa, Italy; 156 Department of Physics and Astronomy, University of Pittsburgh, Pittsburgh, PA USA; 157 Laboratório de Instrumentação e Física Experimental de Partículas -LIP, Lisbon, Portugal; 158 Faculdade de Ciências, Universidade de Lisboa, Lisbon, Portugal; 159 Department of Physics, University of Coimbra, Coimbra, Portugal; 160 Centro de Física Nuclear da Universidade de Lisboa, Lisbon, Portugal; 161 Departamento de Fisica, Universidade do Minho, Braga, Portugal; 162 Departamento de Fisica Teorica y del Cosmos and CAFPE, Universidad de Granada, Granada, Spain; 163 Dep Fisica and CEFITEC of Faculdade de Ciencias e Tecnologia, Universidade Nova de Lisboa, Caparica, Portugal; 164 Institute of Physics, Academy of Sciences of the Czech Republic, Prague, Czech Republic; 165 Czech Technical University in Prague, Prague, Czech Republic; 166 Faculty of Mathematics and Physics, Charles University in Prague, Prague, Czech Republic; 167 State Research Center Institute for High Energy Physics (Protvino), NRC KI, Russia; 168 Particle Physics Department, Rutherford Appleton Laboratory, Didcot, UK; 169 INFN Sezione di Roma, Rome, Italy; 170 Dipartimento di Fisica, Sapienza Università di Roma, Rome, Italy; 171 INFN Sezione di Roma Tor Vergata, Rome, Italy; 172 Dipartimento di Fisica, Università di Roma Tor Vergata, Rome, Italy; 173 INFN Sezione di Roma Tre, Rome, Italy; 174 Dipartimento di Matematica e Fisica, Università Roma Tre, Rome, Italy; 175 Faculté des Sciences Ain Chock, Réseau Universitaire de Physique des Hautes Energies-Université Hassan II, Casablanca, Morocco; 176 Centre National de l’Energie des Sciences Techniques Nucleaires, Rabat, Morocco; 177 Faculté des Sciences Semlalia, Université Cadi Ayyad, LPHEA-Marrakech, Marrakech, Morocco; 178 Faculté des Sciences, Université Mohamed Premier and LPTPM, Oujda, Morocco; 179 Faculté des Sciences, Université Mohammed V, Rabat, Morocco; 180 DSM/IRFU (Institut de Recherches sur les Lois Fondamentales de l’Univers), CEA Saclay (Commissariat à l’Energie Atomique et aux Energies Alternatives), Gif-sur-Yvette, France; 181 Santa Cruz Institute for Particle Physics, University of California Santa Cruz, Santa Cruz, CA USA; 182 Department of Physics, University of Washington, Seattle, WA USA; 183 School of Physics, Shandong University, Shandong, China; 184 Department of Physics and Astronomy, Shanghai Key Laboratory for Particle Physics and Cosmology, Shanghai Jiao Tong University, (also affiliated with PKU-CHEP), Shanghai, China; 185 Department of Physics and Astronomy, University of Sheffield, Sheffield, UK; 186 Department of Physics, Shinshu University, Nagano, Japan; 187 Fachbereich Physik, Universität Siegen, Siegen, Germany; 188 Department of Physics, Simon Fraser University, Burnaby, BC Canada; 189 SLAC National Accelerator Laboratory, Stanford, CA USA; 190 Faculty of Mathematics, Physics and Informatics, Comenius University, Bratislava, Slovak Republic; 191 Department of Subnuclear Physics, Institute of Experimental Physics of the Slovak Academy of Sciences, Kosice, Slovak Republic; 192 Department of Physics, University of Cape Town, Cape Town, South Africa; 193 Department of Physics, University of Johannesburg, Johannesburg, South Africa; 194 School of Physics, University of the Witwatersrand, Johannesburg, South Africa; 195 Department of Physics, Stockholm University, Stockholm, Sweden; 196 The Oskar Klein Centre, Stockholm, Sweden; 197 Physics Department, Royal Institute of Technology, Stockholm, Sweden; 198 Departments of Physics and Astronomy and Chemistry, Stony Brook University, Stony Brook, NY USA; 199 Department of Physics and Astronomy, University of Sussex, Brighton, UK; 200 School of Physics, University of Sydney, Sydney, NSW Australia; 201 Institute of Physics, Academia Sinica, Taipei, Taiwan; 202 Department of Physics, Technion: Israel Institute of Technology, Haifa, Israel; 203 Raymond and Beverly Sackler School of Physics and Astronomy, Tel Aviv University, Tel Aviv, Israel; 204 Department of Physics, Aristotle University of Thessaloniki, Thessaloniki, Greece; 205 International Center for Elementary Particle Physics and Department of Physics, The University of Tokyo, Tokyo, Japan; 206 Graduate School of Science and Technology, Tokyo Metropolitan University, Tokyo, Japan; 207 Department of Physics, Tokyo Institute of Technology, Tokyo, Japan; 208 Department of Physics, University of Toronto, Toronto, ON Canada; 209 TRIUMF, Vancouver, BC Canada; 210 Department of Physics and Astronomy, York University, Toronto, ON Canada; 211 Faculty of Pure and Applied Sciences, and Center for Integrated Research in Fundamental Science and Engineering, University of Tsukuba, Tsukuba, Japan; 212 Department of Physics and Astronomy, Tufts University, Medford, MA USA; 213 Department of Physics and Astronomy, University of California Irvine, Irvine, CA USA; 214 INFN Gruppo Collegato di Udine, Sezione di Trieste, Udine, Italy; 215 ICTP, Trieste, Italy; 216 Dipartimento di Chimica Fisica e Ambiente, Università di Udine, Udine, Italy; 217 Department of Physics and Astronomy, University of Uppsala, Uppsala, Sweden; 218 Department of Physics, University of Illinois, Urbana, IL USA; 219 Instituto de Fisica Corpuscular (IFIC) and Departamento de Fisica Atomica, Molecular y Nuclear and Departamento de Ingeniería Electrónica and Instituto de Microelectrónica de Barcelona (IMB-CNM), University of Valencia and CSIC, Valencia, Spain; 220 Department of Physics, University of British Columbia, Vancouver, BC Canada; 221 Department of Physics and Astronomy, University of Victoria, Victoria, BC Canada; 222 Department of Physics, University of Warwick, Coventry, UK; 223 Waseda University, Tokyo, Japan; 224 Department of Particle Physics, The Weizmann Institute of Science, Rehovot, Israel; 225 Department of Physics, University of Wisconsin, Madison, WI USA; 226 Fakultät für Physik und Astronomie, Julius-Maximilians-Universität, Würzburg, Germany; 227 Fakultät für Mathematik und Naturwissenschaften, Fachgruppe Physik, Bergische Universität Wuppertal, Wuppertal, Germany; 228 Department of Physics, Yale University, New Haven, CT USA; 229 Yerevan Physics Institute, Yerevan, Armenia; 230 Centre de Calcul de l’Institut National de Physique Nucléaire et de Physique des Particules (IN2P3), Villeurbanne, France; 231 CERN, 1211 Geneva 23, Switzerland

## Abstract

A measurement of the $t\bar{t}Z$ and $t\bar{t}W$ production cross sections in final states with either two same-charge muons, or three or four leptons (electrons or muons) is presented. The analysis uses a data sample of proton–proton collisions at $\sqrt{s} = 13$ TeV recorded with the ATLAS detector at the Large Hadron Collider in 2015, corresponding to a total integrated luminosity of 3.2 fb$^{-1}$. The inclusive cross sections are extracted using likelihood fits to signal and control regions, resulting in $\sigma _{t\bar{t}Z} = 0.9 \pm 0.3$ pb and $\sigma _{t\bar{t}W} = 1.5 \pm 0.8$ pb, in agreement with the Standard Model predictions.

## Introduction

At the Large Hadron Collider (LHC), top quarks are copiously produced in quark–antiquark pairs ($t\bar{t}$). This process has been extensively studied in proton–proton collisions at 7 and $8\,\text {TeV}$, and recently at $13\,\text {TeV}$ [1, 2] centre-of-mass energy. Measurements of the associated production of $t\bar{t}$ with a *Z* boson ($t\bar{t} Z$) allow the extraction of information about the neutral-current coupling of the top quark. The production rate of a top-quark pair with a massive vector boson could be altered in the presence of physics beyond the Standard Model (SM), such as vector-like quarks [3, 4], strongly coupled Higgs bosons [5] or technicolour [6–10], and therefore the measurements of $\sigma _{t\bar{t} Z}$ and $\sigma _{t\bar{t} W}$ are important checks of the validity of the SM at this new energy regime. The $t\bar{t} Z$ and $t\bar{t} W$ processes have been established by ATLAS [11] and CMS [12] using the Run-1 dataset at $\sqrt{s} = 8\,\text {TeV}$, with measured cross sections compatible with the SM prediction and having uncertainties of $\sim \!\!30\%$. At $\sqrt{s} = 13\,\text {TeV}$, the SM cross sections of the $t\bar{t} Z$ and $t\bar{t} W$ processes increase by factors of 3.5 and 2.4, respectively, compared to $\sqrt{s} = 8\,\text {TeV}$. The cross sections, computed at next-to-leading-order (NLO) QCD precision, using MadGraph5_aMC@NLO (referred to in the following as MG5_aMC), are $\sigma _{t\bar{t} Z} = 0.84\,\text {pb}$ and $\sigma _{t\bar{t} W} = 0.60\,\text {pb}$ with an uncertainty of $\sim \!\!12\%$ [13, 14], primarily due to higher-order corrections, estimated by varying the renormalisation and factorisation scales.

This paper presents measurements of the $t\bar{t} Z$ and $t\bar{t} W$ cross sections using $3.2\,\text{ fb }^{-1}$ of proton–proton (*pp*) collision data at $\sqrt{s} = 13\,\text {TeV}$ collected by the ATLAS detector in 2015. The final states of top-quark pairs produced in association with a *Z* or a *W* boson comprise up to four isolated, prompt leptons.^1^ Decay modes with two same-sign (SS) charged muons, or three or four leptons are considered in this analysis. The analysis strategy follows the strategy adopted for the $8\,\text {TeV}$ dataset [11], excluding the lower sensitivity SS dilepton channels. Table 1 lists the analysis channels and the targeted decay modes of the $t\bar{t} Z$ and $t\bar{t} W$ processes. Each channel is divided into multiple analysis regions in order to enhance the sensitivity to the signal. Simultaneous fits are performed to the signal regions and selected control regions in order to extract the cross sections for $t\bar{t} Z$ and $t\bar{t} W$ production. Additional validation regions are defined to check that the background estimate agrees with the data and are not used in the fit.

**Table 1:** List of $t\bar{t} W$ and $t\bar{t} Z$ decay modes and analysis channels targeting them

Process	$t\bar{t}$ decay	Boson decay	Channel
$t\bar{t} W ^{\pm }$	$(\mu ^{\pm }\nu b) (q\bar{q} b)$	$\mu ^{\pm }\nu$	SS dimuon
	$(\ell ^{\pm }\nu b) (\ell ^{\mp }\nu b)$	$\ell ^{\pm }\nu$	Trilepton
$t\bar{t} Z$	$(\ell ^{\pm }\nu b) (q\bar{q} b)$	$\ell ^{+}\ell ^{-}$	Trilepton
	$(\ell ^{\pm }\nu b) (\ell ^{\mp } \nu b)$	$\ell ^{+}\ell ^{-}$	Tetralepton

## The ATLAS detector

The ATLAS detector [15] consists of four main subsystems: an inner tracking system, electromagnetic (EM) and hadronic calorimeters, and a muon spectrometer (MS). The inner detector (ID) consists of a high-granularity silicon pixel detector, including the newly installed Insertable B-Layer [16], which is the innermost layer of the tracking system, and a silicon microstrip tracker, together providing precision tracking in the pseudorapidity^2^ range $|\eta |<2.5$ and of a transition radiation tracker covering $|\eta |<2.0$. All the systems are immersed in a 2T magnetic field provided by a superconducting solenoid. The EM sampling calorimeter uses lead and liquid argon (LAr) and is divided into barrel ($|\eta |<1.475$) and endcap ($1.375<|\eta |<3.2$) regions. Hadron calorimetry is provided by a steel/scintillator-tile calorimeter, segmented into three barrel structures, in the range $|\eta |<1.7$, and by two copper/LAr hadronic endcap calorimeters that cover the region $1.5<|\eta |<3.2$. The solid angle coverage is completed with forward copper/LAr and tungsten/LAr calorimeter modules, optimised for EM and hadronic measurements respectively, covering the region $3.1<|\eta |<4.9$. The muon spectrometer measures the deflection of muon tracks in the range $|\eta |<2.7$ using multiple layers of high-precision tracking chambers located in toroidal magnetic fields. The field integral of the toroids ranges between 2.0 and 6.0Tm for most of the detector. The muon spectrometer is also instrumented with separate trigger chambers covering $|\eta |<2.4$. A two-level trigger system, using custom hardware followed by a software-based trigger level, is used to reduce the event rate to an average of around 1 kHz for offline storage.

## Data and simulated event samples

The data were collected with the ATLAS detector during 2015 with a bunch spacing of 25 ns and a mean number of 14 *pp* interactions per bunch crossing (pile-up). With strict data-quality requirements, the integrated luminosity considered corresponds to $3.2\,\text{ fb }^{-1}$ with an uncertainty of $2.1\%$ [17].

Monte Carlo simulation samples (MC) are used to model the expected signal and background distributions in the different control, validation and signal regions described below. The heavy-flavour decays involving $b-$ and $c-$quarks, particularly important to this measurement, are modelled using the EvtGen  [18] program, except for processes modelled using the Sherpa generator. In all samples the top-quark mass is set to $172.5\,\text {GeV}$ and the Higgs boson mass is set to $125\,\text {GeV}$. The response of the detector to stable^3^ particles is emulated by a dedicated simulation [19] based either fully on Geant  [20] or on a faster parameterisation [21] for the calorimeter response and Geant for other detector systems. To account for additional *pp* interactions from the same and close-by bunch crossings, a set of minimum-bias interactions generated using Pythia v8.210 [22], referred to as Pythia 8 in the following, with the A2 [23] set of tuned MC parameters (A2 tune) is superimposed on the hard-scattering events. In order to reproduce the same pile-up levels present in the data, the distribution of the number of additional *pp* interactions in the MC samples is reweighted to match the one in the data. All samples are processed through the same reconstruction software as the data. Simulated events are corrected so that the object identification, reconstruction and trigger efficiencies, energy scales and energy resolutions match those determined from data control samples.

The associated production of a top-quark pair with one or two vector bosons is generated at leading order (LO) with MG5_aMC interfaced to Pythia 8, with up to two ($t\bar{t} W$), one ($t\bar{t} Z$) or no ($t\bar{t} WW$) extra partons included in the matrix elements. The $\gamma ^{*}$ contribution and the $Z/\gamma ^{*}$ interference are included in the $t\bar{t} Z$ samples. The A14  [24] set of tuned MC parameters (A14 tune) is used together with the NNPDF2.3LO parton distribution function (PDF) set [25]. The samples are normalised using cross sections computed at NLO in QCD [26].

The *t*-channel production of a single top quark in association with a *Z* boson ($tZ$) is generated using MG5_aMC interfaced with Pythia v6.427 [27], referred to as Pythia 6 in the following, with the CTEQ6L1 PDF [28] set and the Perugia2012 [29] set of tuned MC parameters at NLO in QCD. The $Z/\gamma ^{*}$ interference is included, and the four-flavour scheme is used in the computation.

The $Wt$-channel production of a single top quark together with a *Z* boson ($tWZ$) is generated with MG5_aMC and showered with Pythia 8, using the NNPDF3.0NLO PDF set [30] and the A14 tune. The generation is performed at NLO in QCD using the five-flavour scheme. Diagrams containing a top-quark pair are removed to avoid overlap with the $t\bar{t} Z$ process.

Diboson processes with four charged leptons ($4\ell$), three charged leptons and one neutrino ($\ell \ell \ell \nu$) or two charged leptons and two neutrinos ($\ell \ell \nu \nu$) are simulated using the Sherpa 2.1 generator [31]. The matrix elements include all diagrams with four electroweak vertices. They are calculated for up to one ($4\ell , \ell \ell \nu \nu$) or no additional partons ($\ell \ell \ell \nu$) at NLO and up to three partons at LO using the Comix  [32] and OpenLoops  [33] matrix element generators and merged with the Sherpa parton shower using the ME+PS@NLO prescription [34]. The CT10nlo PDF set [35] is used in conjunction with a dedicated parton-shower tuning developed by the Sherpa authors. The NLO cross sections calculated by the generator are used to normalise diboson processes. Alternative diboson samples are simulated using the Powheg-Box v2 [36] generator, interfaced to the Pythia 8 parton shower model, and for which the CT10nlo PDF set is used in the matrix element, while the CTEQ6L1 PDF set is used for the parton shower along with the AZNLO [37] set of tuned MC parameters.

The production of three massive vector bosons with subsequent leptonic decays of all three bosons is modelled at LO with the Sherpa 2.1 generator and the CT10 PDF set [35]. Up to two additional partons are included in the matrix element at LO and the full NLO accuracy is used for the inclusive process.

Electroweak processes involving the vector-boson scattering (VBS) diagram and producing two same-sign leptons, two neutrinos and two partons are modelled using Sherpa 2.1 at LO accuracy and the CT10 PDF set. Processes of orders four and six in the electroweak coupling constant are considered, and up to one additional parton is included in the matrix element.

For the generation of $t\bar{t}$ events and $Wt$-channel single-top-quark events the Powheg-Box v2 generator is used with the CT10 PDF set. The parton shower and the underlying event are simulated using Pythia 6 with the CTEQ6L1 PDF set and the corresponding Perugia2012 tune. The $t\bar{t}$ samples are normalised to their next-to-next-to-leading-order (NNLO) cross-section predictions, including soft-gluon resummation to next-to-next-to-leading-log order, as calculated with the Top++2.0 program (see Ref. [38] and references therein). For more efficient sample generation, the $t\bar{t}$ sample is produced by selecting only true dilepton events in the final state. Moreover, an additional dilepton $t\bar{t}$ sample requiring a *b*-hadron not coming from top-quark decays is generated after *b*-jet selection. Diagram removal is employed to remove the overlap between $t\bar{t}$ and $Wt$  [39].

Samples of $t\bar{t}$ events produced in association with a Higgs boson ($t\bar{t} H$) are generated using NLO matrix elements in MG5_aMC with the CT10NLO PDF set and interfaced with Pythia 8 for the modelling of the parton shower. Higgs boson production via gluon–gluon fusion (ggF) and vector boson fusion (VBF) is generated using the Powheg-Box v2 generator with CT10 PDF set. The parton shower and underlying event are simulated using Pythia 8 with the CTEQ6L1 PDF set and AZNLO tune. Higgs boson production with a vector boson is generated at LO using Pythia 8 with the CTEQ6L1 PDF. All Higgs boson samples are normalised using theoretical calculations of Ref. [40].

Events containing *Z* or *W* bosons with associated jets, referred to as *Z*+jets and *W*+jets in the following, are simulated using the Sherpa 2.1 generator. Matrix elements are calculated for up to two partons at NLO and four partons at LO. The CT10 PDF set is used in conjunction with a dedicated parton-shower tuning developed by the Sherpa authors [31]. The *Z* / *W*+jets samples are normalised to the NNLO cross sections [41–44]. Alternative *Z* / *W*+jets samples are simulated using MG5_aMC at LO interfaced to the Pythia 8 parton shower model. The A14 tune is used together with the NNPDF2.3LO PDF set.

The SM production of three and four top quarks is generated at LO with MG5_aMC+Pythia 8, using the A14 tune together with the NNPDF2.3LO PDF set. The samples are normalised using cross sections computed at NLO [45, 46].

## Object reconstruction

The final states of interest in this analysis contain electrons, muons, jets, *b*-jets and missing transverse momentum.

Electron candidates [47] are reconstructed from energy deposits (clusters) in the EM calorimeter that are associated with reconstructed tracks in the inner detector. The electron identification relies on a likelihood-based selection [48, 49]. Electrons are required to pass the “medium” likelihood identification requirements described in Ref. [49]. These include requirements on the shapes of the electromagnetic shower in the calorimeter as well as tracking and track-to-cluster matching quantities. The electrons are also requirement to have transverse momentum $p_{\text {T}} > 7\,\text {GeV}$ and $|\eta _\mathrm {cluster}| < 2.47$, where $\eta _\mathrm {cluster}$ is the pseudorapidity of the calorimeter energy deposit associated with the electron candidate. Candidates in the EM calorimeter barrel/endcap transition region $1.37< |\eta _\mathrm {cluster}| < 1.52$ are excluded.

Muon candidates are reconstructed from a fit to track segments in the various layers of the muon spectrometer, matched with tracks identified in the inner detector. Muons are required to have $p_{\text {T}} > 7\,\text {GeV}$ and $|\eta | < 2.4$ and to pass the “medium” identification requirements defined in Ref. [50]. The medium requirement includes selections on the numbers of hits in the ID and MS as well as a compatibility requirement between momentum measurements in the ID and MS. It provides a high efficiency and purity of selected muons. Electron candidates sharing a track with a muon candidate are removed.

To reduce the non-prompt lepton background from hadron decays or jets misidentified as leptons (labelled as “fake leptons” throughout this paper), electron and muon candidates are required to be isolated. The total sum of track transverse momenta in a surrounding cone of size $\min (10\,\text {GeV}/p_{\text {T}}, r_{e,\mu })$, excluding the track of the candidate from the sum, is required to be less than $6\%$ of the candidate $p_{\text {T}}$, where $r_e = 0.2$ and $r_{\mu } = 0.3$. In addition, the sum of the cluster transverse energies in the calorimeter within a cone of size $\Delta R_{\eta } \equiv \sqrt{(\Delta \eta )^2 + (\Delta \phi )^2} = 0.2$ of any electron candidate, excluding energy deposits of the candidate itself, is required to be less than $6\%$ of the candidate $p_{\text {T}}$.

For both electrons and muons, the longitudinal impact parameter of the associated track with respect to the primary vertex,^4^ $z_{0}$, is required to satisfy $|z_0 \sin \theta |<0.5$ mm. The significance of the transverse impact parameter $d_0$ is required to satisfy $|d_0|/\sigma (d_0)<5$ for electrons and $|d_0|/\sigma (d_0)<3$ for muons, where $\sigma (d_0)$ is the uncertainty in $d_0$.

Jets are reconstructed using the anti-$k_t$ algorithm [51, 52] with radius parameter $R = 0.4$, starting from topological clusters in the calorimeters [53]. The effect of pile-up on jet energies is accounted for by a jet-area-based correction [54] and the energy resolution of the jets is improved by using global sequential corrections [55]. Jets are calibrated to the hadronic energy scale using *E*- and $\eta$-dependent calibration factors based on MC simulations, with in-situ corrections based on Run-1 data [56, 57] and checked with early Run-2 data [58]. Jets are accepted if they fulfil the requirements $p_{\text {T}} > 25\,\text {GeV}$ and $|\eta | < 2.5$. To reduce the contribution from jets associated with pile-up, jets with $p_{\text {T}} < 60\,\text {GeV}$ and $|\eta | < 2.4$ are required to satisfy pile-up rejection criteria (JVT), based on a multivariate combination of track-based variables  [59].

Jets are *b*-tagged as likely to contain *b*-hadrons using the MV2c20 algorithm, a multivariate discriminant making use of the long lifetime, large decay multiplicity, hard fragmentation and high mass of *b*-hadrons [60]. The average efficiency to correctly tag a *b*-jet is approximately $77\%$, as determined in simulated $t\bar{t}$ events, but it varies as a function of $p_{\text {T}}$ and $\eta$. In simulation, the tagging algorithm gives a rejection factor of about 130 against light-quark and gluon jets, and about 4.5 against jets containing charm quarks [61]. The efficiency of *b*-tagging in simulation is corrected to that in data using a $t\bar{t}$-based calibration using Run-1 data [62] and validated with Run-2 data [63].

The missing transverse momentum $\mathbf {p}^\mathrm {miss}_\mathrm {T}$, with magnitude $E_{\text {T}}^{\text {miss}}$, is a measure of the transverse momentum imbalance due to particles escaping detection. It is computed [64] as the negative sum of the transverse momenta of all electrons, muons and jets and an additional soft term. The soft term is constructed from all tracks that are associated with the primary vertex but not with any physics object. In this way, the $E_{\text {T}}^{\text {miss}}$ is adjusted for the best calibration of the jets and the other identified physics objects above, while maintaining pile-up independence in the soft term [65, 66].

To prevent double-counting of electron energy deposits as jets, the closest jet within $\Delta R_y = 0.2$ of a reconstructed electron is removed, where $\Delta R_y \equiv \sqrt{(\Delta y)^2 + (\Delta \phi )^2}$. If the nearest jet surviving the above selection is within $\Delta R_y = 0.4$ of an electron, the electron is discarded to ensure that selected electrons are sufficiently separated from nearby jet activity. To reduce the background from muons originating from heavy-flavour particle decays inside jets, muons are removed if they are separated from the nearest jet by $\Delta R_y < 0.4$. However, if this jet has fewer than three associated tracks, the muon is kept and the jet is removed instead; this avoids an inefficiency for high-energy muons undergoing significant energy loss in the calorimeter.

## Event selection and background estimation

Only events collected using single-electron or single-muon triggers are accepted. The trigger thresholds, $p_{\text {T}} > 24\,\text {GeV}$ for electrons and $p_{\text {T}} > 20\,\text {GeV}$ for muons, are set to be almost fully efficient for reconstructed leptons with $p_{\text {T}} > 25\,\text {GeV}$. Events are required to have at least one reconstructed primary vertex. In all selections considered, at least one reconstructed lepton with $p_{\text {T}} > 25\,\text {GeV}$ is required to match ($\Delta R_{\eta } < 0.15$) a lepton with the same flavour reconstructed by the trigger algorithm. Three channels are defined based on the number of reconstructed leptons, which are sorted according to their transverse momentum in decreasing order.

Background events containing well-identified prompt leptons are modelled by simulation. The normalisations for the *WZ* and *ZZ* processes are taken from data control regions and included in the fit. The yields in these data control regions are extrapolated to the signal regions using simulation. Systematic uncertainties in the extrapolation are taken into account in the overall uncertainty in the background estimate.

Background sources involving one or more fake leptons are modelled using data events from control regions. For the same-sign dimuon ($2\mu$-SS) analysis and the trilepton analysis the fake-lepton background is estimated using the matrix method [67], where any combination of fake leptons among the selected leptons is considered. However, compared to Ref. [67], the real- and fake-lepton efficiencies used by the matrix method are estimated in a different way in this measurement. The lepton efficiencies are measured by applying the matrix method in control regions, where the lepton efficiencies are extracted in a likelihood fit as free parameters using the matrix method as model, assuming Poisson statistics, and assuming that events with two fake leptons are negligible. In this way the parameters are by construction the actual parameters of the matrix model itself, instead of relying on external lepton efficiency measurements, which are not guaranteed to be fully consistent with the matrix model. The control regions are defined in dilepton events, separately for *b*-tagged and *b*-vetoed events to take into account the different fake-lepton efficiencies depending on whether the source is a light-flavour jet or a heavy-flavour jet. The real-lepton efficiencies are measured in inclusive opposite-sign events, and fake-lepton efficiencies in events with same-sign leptons and $E_{\text {T}}^{\text {miss}} >40\,\text {GeV}$ (for *b*-tagged events $E_{\text {T}}^{\text {miss}} >20\,\text {GeV}$), after subtracting the estimated contribution from events with misidentification of the charge of a lepton (referred to as “charge-flip” in the following), and excluding the same-sign dimuon signal region. The charge-flip events are subtracted using simulation. The extracted fake-lepton efficiencies are found to be compatible with fake-lepton efficiencies from a fully data-driven procedure where the charge-flip events are estimated from data. For the tetralepton channel, the contribution from backgrounds containing fake leptons is estimated from simulation and corrected with scale factors determined in control regions.

The full selection requirements and the background evaluation strategies in the different channels are described below.

### Same-sign dimuon analysis

The same-sign dimuon signal region targets the $t\bar{t} W$ process and has the highest sensitivity among all same-sign dilepton regions [11]. The main reason for this is that electrons have a much larger charge misidentification probability, inducing a significant background from top-quark pairs. Events are required to have two muon candidates with the same charge and $p_{\text {T}} >25\,\text {GeV}$, $E_{\text {T}}^{\text {miss}} > 40\,\text {GeV}$, the scalar sum of the $p_{\text {T}}$ of selected leptons and jets, $H_{\text {T}}$, above $240\,\text {GeV}$, and at least two *b*-tagged jets. Events containing additional leptons (with $p_{\text {T}} >7\,\text {GeV}$) are vetoed.

**Fig. 1:**
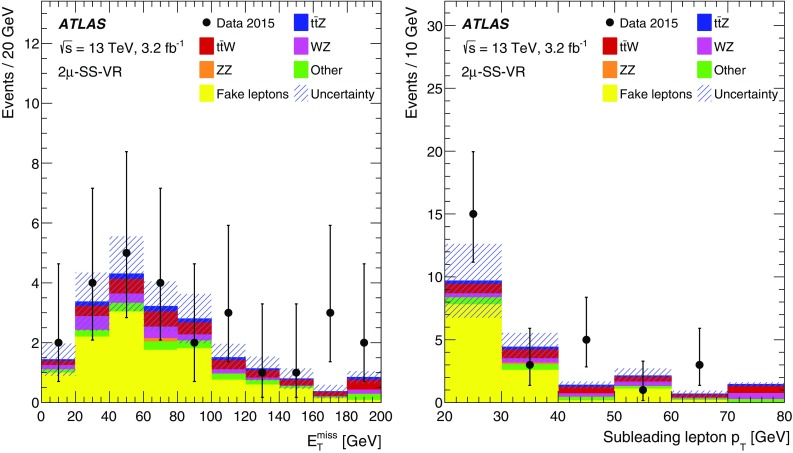
The (*left*) $E_{\text {T}}^{\text {miss}}$ and (*right*) subleading lepton $p_{\text {T}}$ distributions shown for the *b*-tagged $2\mu$-SS channel where the signal region requirements on subleading lepton $p_{\text {T}}$, number of *b*-tags, and $E_{\text {T}}^{\text {miss}}$ are relaxed. The shaded band represents the total uncertainty. The background denoted ‘Other’ contains other SM processes producing two same-sign prompt leptons. The last bin in each of the distributions includes the overflow

The dominant background in the $2\mu$-SS region arises from events containing fake leptons, where the main source is $t\bar{t}$ events. Backgrounds from the production of prompt leptons with correctly identified charge come primarily from *WZ* production, but the relative contribution of this background is small compared to the fake-lepton background. The charge-flip background is negligible in this signal region, as the probability of misidentifying the charge of a muon in the relevant $p_{\text {T}}$ range is negligible. For the validation of the fake-lepton background estimate a region is defined based on the signal region selection but omitting the $E_{\text {T}}^{\text {miss}}$ requirement, reducing the $p_{\text {T}}$ threshold of the subleading lepton to $20\,\text {GeV}$ and requiring at least one *b*-tagged jet. The distributions of $E_{\text {T}}^{\text {miss}}$ and subleading lepton $p_{\text {T}}$ in this validation region ($2\mu$-SS-VR) are shown in Fig. 1. The expected numbers of events in the $2\mu$-SS signal region are shown in Table 4. Nine events are observed in data for this signal region.

### Trilepton analysis

Four signal regions with exactly three leptons are considered. The first three are sensitive to $t\bar{t} Z$; each of these requires an opposite-sign same-flavour (OSSF) pair of leptons whose invariant mass is within $10\,\text {GeV}$ of the *Z* boson mass. The signal regions are categorised by their jet and *b*-jet multiplicities and have different signal-to-background ratios. In the 3$\ell$-Z-1b4j region, at least four jets are required, exactly one of which is *b*-tagged. In the 3$\ell$-Z-2b3j region, exactly three jets with at least two *b*-tagged jets are required. In the 3$\ell$-Z-2b4j region, at least four jets are required, of which at least two are *b*-tagged.

In the 3$\ell$-noZ-2b region at least two and at most four jets are required, of which at least two are *b*-tagged, no OSSF lepton pair is allowed in the *Z* boson mass window, and the sum of the lepton charges must be ±1. This region primarily targets the $t\bar{t} W$ process but also has a sizeable $t\bar{t} Z$ contribution.

The signal region definitions for the trilepton channel are summarised in Table 2, while the expected numbers of events in the signal regions are shown in Table 4. The dominant backgrounds in the 3$\ell$-Z-1b4j, 3$\ell$-Z-2b3j and 3$\ell$-Z-2b4j signal regions arise from *Z*+jets production with a fake lepton, diboson production and the production of a single top quark in association with a *Z* boson.

**Table 2:** Summary of event selections in the trilepton signal regions

Variable	3$\ell$-Z-1b4j	3$\ell$-Z-2b3j	3$\ell$-Z-2b4j	3$\ell$-noZ-2b
Leading leptons *p*_T_	>25 GeV	>25 GeV	>25 GeV	>25 GeV
Other leptons’ *p*_T_	>20 GeV	>20 GeV	>20 GeV	>20 GeV
Sum of leptons’ charges	±1	±1	±1	±1
OSSF $|m_{\ell \ell } - m_Z|$	<10 GeV	<10 GeV	<10 GeV	<10 GeV
$n_{\mathrm {jets}}$	$\ge 4$	3	$\ge 4$	$\ge 2$ and $\le 4$
$n_{b{\mathrm {-jets}}}$	1	$\ge 2$	$\ge 2$	$\ge 2$

**Fig. 2:**
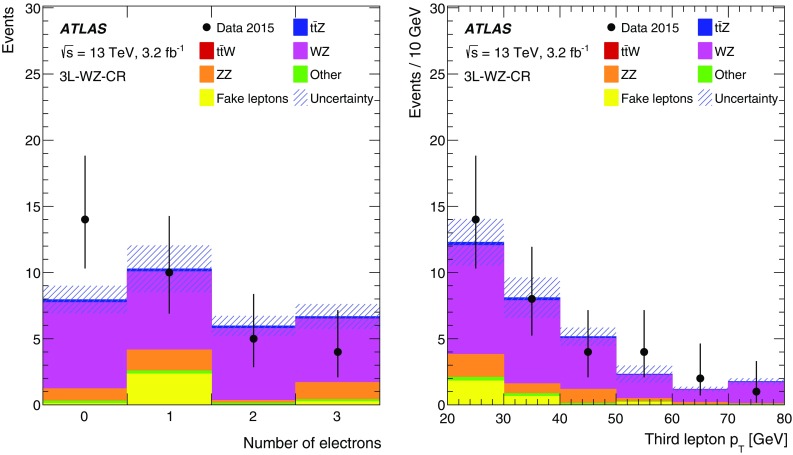
Distributions of (*left*) the number of electrons and (*right*) the third-lepton $p_{\text {T}}$ in the 3$\ell$-WZ-CR control region before the fit. The background denoted ‘Other’ contains other SM processes producing three prompt leptons. The shaded band represents the total uncertainty. The last bin of the distribution shown in the right panel includes the overflow

A control region is used to constrain the normalisation of the *WZ* background in data. Exactly three leptons are required, at least one pair of which must be an OSSF pair with an invariant mass within $10\,\text {GeV}$ of the *Z* boson mass. There must be exactly three jets, none of which pass the *b*-tagging requirement. With these requirements, the expected $t\bar{t} Z$ signal contribution is roughly 1% of the total number of events. This region is referred to as 3$\ell$-WZ-CR and it is included in the fit. Distributions comparing data and SM prediction are shown in Fig. 2.

**Fig. 3:**
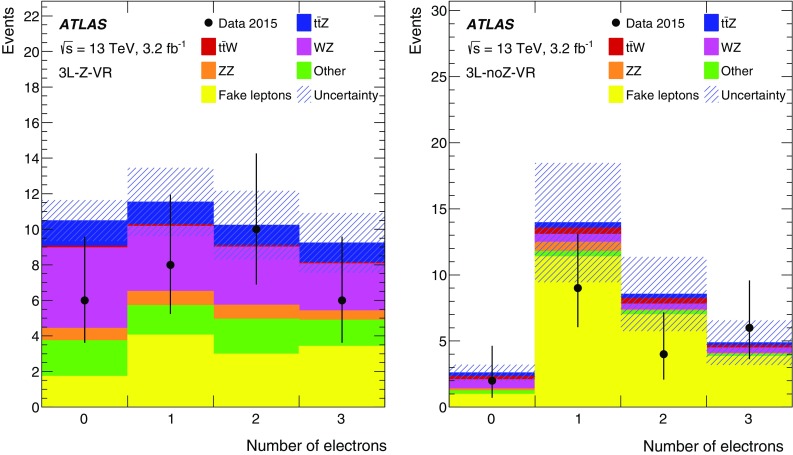
Distributions of the number of electrons in the (*left*) $3\ell$-Z-VR and (*right*) $3\ell$-noZ-VR validation regions, shown before the fit. The background denoted ‘Other’ contains other SM processes producing three prompt leptons. The shaded band represents the total uncertainty

Two background validation regions are defined for the trilepton channel. In the first region, $3\ell$-Z-VR, the presence of two OSSF leptons with an invariant mass within $10\,\text {GeV}$ of the mass of the *Z* boson is required. The region requires the events to have at most three jets where exactly one is *b*-tagged, or exactly two jets where both jets are *b*-tagged. The main backgrounds are *WZ* production and *Z*+jets events with fake leptons. In the second region, $3\ell$-noZ-VR, events with such a pair of leptons are vetoed. This region requires the events to have at most three jets where exactly one is *b*-tagged, and it is dominated by the fake-lepton background from top-quark pair production. Neither validation region is used in the fit. The distributions of the number of electrons in each of the two validation regions are shown in Fig. 3, demonstrating that data and background modelling are in good agreement within statistical uncertainties.

**Fig. 4:**
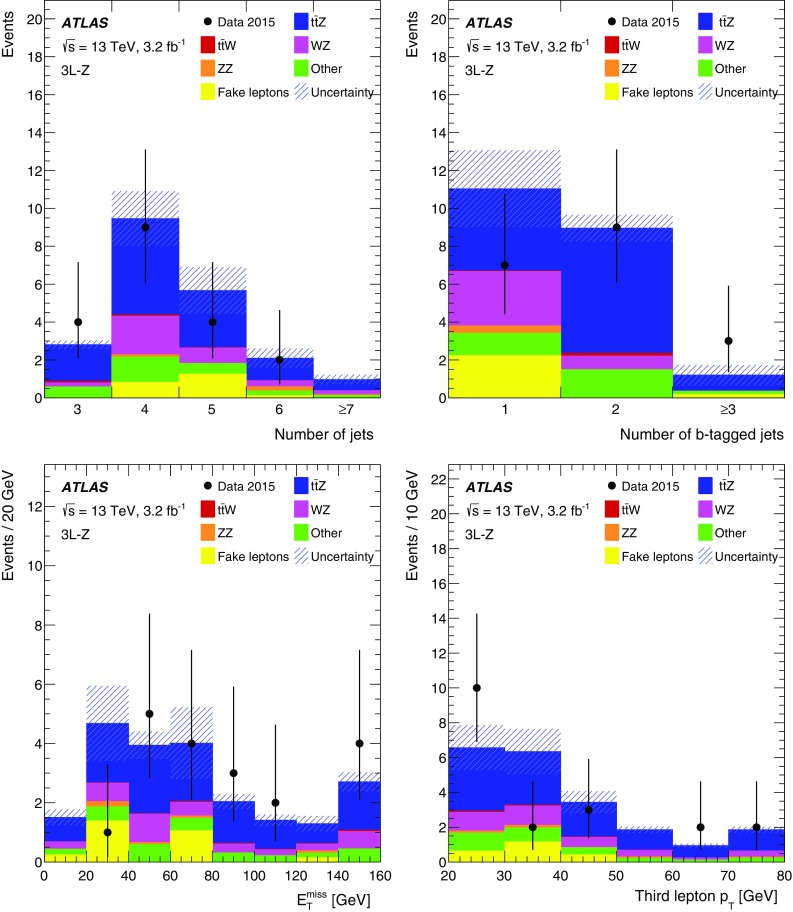
Distributions of (*top left*) the number of jets, (*top right*) the number of *b*-tagged jets, (*bottom left*) the missing transverse momentum and (*bottom right*) the third-lepton $p_{\text {T}}$, for events contained in any of the three signal regions 3$\ell$-Z-1b4j, 3$\ell$-Z-2b3j or 3$\ell$-Z-2b4j. The distributions are shown before the fit. The background denoted ‘Other’ contains other SM processes producing three prompt leptons. The shaded band represents the total uncertainty. The last bin in each of the distributions shown in the *bottom panels* includes the overflow

In total, 29 events are observed in the four signal regions. Distributions of the number of jets, number of *b*-tagged jets, missing transverse momentum and transverse momentum of the third lepton are shown in Fig. 4.

### Tetralepton analysis

The tetralepton channel targets the $t\bar{t} Z$ process for the case where both *W* bosons resulting from top-quark decays and the *Z* boson decay leptonically. Events with two pairs of opposite-sign leptons are selected, and at least one pair must be of same flavour. The OSSF lepton pair with reconstructed invariant mass closest to $m_Z$ is attributed to the $Z$ boson decay and denoted in the following by $Z_1$. The two remaining leptons are used to define $Z_2$. Four signal regions are defined according to the relative flavour of the two $Z_2$ leptons, different flavour (DF) or same flavour (SF), and the number of *b*-tagged jets: one, or at least two (1*b*, 2*b*). The signal regions are thus 4$\ell$-DF-1b, 4$\ell$-DF-2b, 4$\ell$-SF-1b and 4$\ell$-SF-2b.

To suppress events with fake leptons in the 1-*b*-tag multiplicity regions, additional requirements on the scalar sum of the transverse momenta of the third and fourth leptons ($p_{\text {T34}}$) are imposed. In the 4$\ell$-SF-1b and 4$\ell$-DF-1b regions, events are required to satisfy $p_{\text {T34}} > {25}\,{\text {GeV}}$ and $p_{\text {T34}} > {35}\,{\text {GeV}}$, respectively. In all regions, the invariant mass of any two reconstructed OS leptons is required to be larger than 10 $\text {GeV}$. The signal region definitions for the tetralepton channel are summarised in Table 3.

**Table 3:** Definitions of the four signal regions in the tetralepton channel. All leptons are required to satisfy $p_{\text {T}} > 7\,\text {GeV}$ and at least one lepton with $p_{\text {T}} > 25\,\text {GeV}$ is required to be trigger matched. The invariant mass of any two reconstructed OS leptons is required to be larger than 10 $\text {GeV}$

Region	$Z_2$ leptons	$p_{\text {T34}}$	$|m_{Z_{2}} - m_Z|$	$E_{\text {T}}^{\text {miss}}$	$n_{b{\mathrm {-tags}}}$
4$\ell$-DF-1b	$e^{\pm }\mu ^{\mp }$	$>{35}{\text {GeV}}$	–	–	1
4$\ell$-DF-2b	$e^{\pm }\mu ^{\mp }$	–	–	–	$\ge 2$
4$\ell$-SF-1b	$e^{\pm }e^{\mp },\mu ^{\pm }\mu ^{\mp }$	$>{25}{\text {GeV}}$	$$\left\{ \begin{array}{ll} {>}10\,\mathrm{{GeV}}&{}{>}40\,\mathrm{{GeV}} \\ {<}10\,\mathrm{{GeV}}&{}{>}80\,\mathrm{{GeV}} \end{array}\right\} $$		1
4$\ell$-SF-2b	$e^{\pm }e^{\mp },\mu ^{\pm }\mu ^{\mp }$	–	$$ \left\{ \begin{array}{ll} {>}10\,\mathrm{{GeV}}&{}-\\ {<}10\,\mathrm{{GeV}}&{}{>}40\,\mathrm{{GeV}} \end{array}\right\} $$		$\ge 2$

**Fig. 5:**
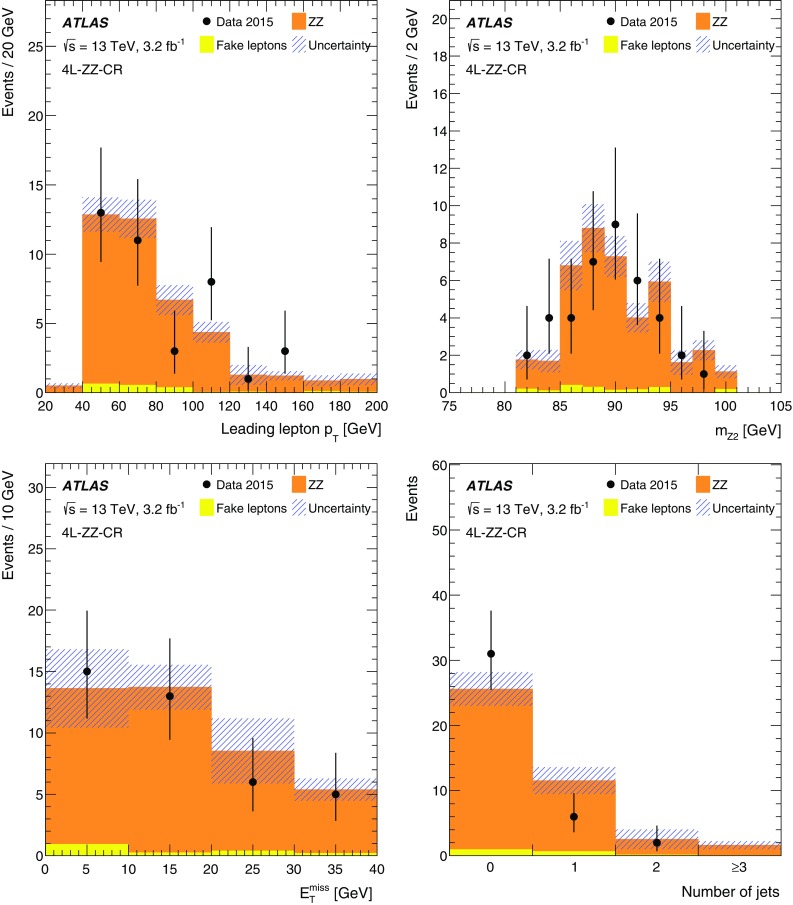
(*Top left*) Leading lepton $p_{\text {T}}$, (*top right*) $m_{Z_2}$, (*bottom left*) missing transverse momentum and (*bottom right*) jet multiplicity distributions in the 4$\ell$-ZZ-CR control region. The distributions are shown before the fit. The shaded band represents the total uncertainty. The last bin of the distribution shown in the *top left panel* includes the overflow

A control region used to constrain the *ZZ* normalisation, referred to as 4$\ell$-ZZ-CR, is included in the fit and is defined to have exactly four reconstructed leptons, a $Z_2$ pair with OSSF leptons, the value of both $m_{Z_1}$ and $m_{Z_2}$ within $10\,\text {GeV}$ of the mass of the *Z* boson, and $E_{\text {T}}^{\text {miss}} <40\,\text {GeV}$. The leading lepton $p_{\text {T}}$, the invariant mass of the $Z_2$ lepton pair, the missing transverse momentum and the jet multiplicity in this control region are shown in Fig. 5, and good agreement is seen between data and prediction.

The contribution from backgrounds containing fake leptons is estimated from simulation and corrected with scale factors determined in two control regions: one region enriched in $t\bar{t}$ events and thus in heavy-flavour jets, and one region enriched in $Z+$jets events, and thus in light-flavour jets. The scale factors are calibrated separately for electron and muon fake-lepton candidates. The scale factors are applied to all MC simulation events with fewer than four prompt leptons according to the number and the flavour of the fake leptons. The $t\bar{t}$ scale factors are applied to MC processes with real top quarks, while for all other processes the $Z+$jets scale factors are applied. Different generators are used when determining the scale factors and when applying them. It is verified that the uncertainties in the scale factors include the differences between these generators.

**Fig. 6:**
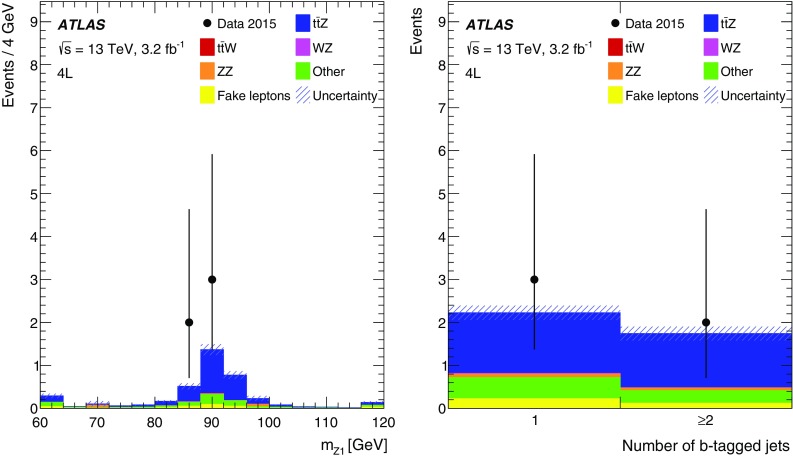
Distributions (*left*) of the invariant mass of the OSSF lepton pair closest to the *Z* boson mass, $m_{Z_1}$, and (*right*) of the number of *b*-tagged jets, for events in the tetralepton signal regions. The distributions are shown before the fit. The background denoted ‘Other’ contains other SM processes producing four prompt leptons. The shaded band represents the total uncertainty. The first and last bin of the distribution shown in the *left panel* include the underflow and overflow, respectively

The expected yields in the signal and control regions in the tetralepton channel are shown in Table 4. Five events are observed in the four signal regions. Figure 6 shows the data superimposed to the expected distributions for all four signal regions combined. Overall the acceptance times efficiency for the $t\bar{t} Z$ and $t\bar{t} W$ processes is 6‰ and 2‰, respectively.

**Table 4:** Expected event yields for signal and backgrounds, and the observed data in all control and signal regions used in the fit to extract the $t\bar{t} Z$ and $t\bar{t} W$ cross sections. The quoted uncertainties in expected event yields represent systematic uncertainties including MC statistical uncertainties. The $tZ$, $tWZ$, $t\bar{t} H$, three- and four-top-quark processes are denoted $t+X$. The *WZ*, *ZZ*, $H \rightarrow ZZ$ (ggF and VBF), *HW* and *HZ* and VBS processes are denoted ‘Bosons’

Region	$t+X$	Bosons	Fake leptons	Total bkg.	$t\bar{t} W$	$t\bar{t} Z$	Data
3$\ell$-WZ-CR	0.52 ± 0.13	26.9 ± 2.2	2.2 ± 1.8	29.5 ± 2.8	0.015 ± 0.004	0.80 ± 0.13	33
4$\ell$-ZZ-CR	$<0.001$	39.5 ± 2.6	1.8 ± 0.6	41.2 ± 2.7	$<0.001$	0.026 ± 0.007	39
$2\mu$-SS	0.94 ± 0.08	0.12 ± 0.05	1.5 ± 1.3	2.5 ± 1.3	2.32 ± 0.33	0.70 ± 0.10	9
3$\ell$-Z-2b4j	1.08 ± 0.25	0.5 ± 0.4	$<0.001$	1.6 ± 0.5	0.065 ± 0.013	5.5 ± 0.7	8
3$\ell$-Z-1b4j	1.14 ± 0.24	3.3 ± 2.2	2.2 ± 1.7	6.7 ± 2.8	0.036 ± 0.011	4.3 ± 0.6	7
3$\ell$-Z-2b3j	0.58 ± 0.19	0.22 ± 0.18	$<0.001$	0.80 ± 0.26	0.083 ± 0.014	1.93 ± 0.28	4
3$\ell$-noZ-2b	0.95 ± 0.11	0.14 ± 0.12	3.6 ± 2.2	4.7 ± 2.2	1.59 ± 0.28	1.45 ± 0.20	10
4$\ell$-SF-1b	0.212 ± 0.032	0.09 ± 0.07	0.113 ± 0.022	0.42 ± 0.08	$<0.001$	0.66 ± 0.09	1
4$\ell$-SF-2b	0.121 ± 0.021	0.07 ± 0.06	0.062 ± 0.012	0.25 ± 0.07	$<0.001$	0.63 ± 0.09	1
4$\ell$-DF-1b	0.25 ± 0.04	0.0131 ± 0.0032	0.114 ± 0.019	0.37 ± 0.04	$<0.001$	0.75 ± 0.10	2
4$\ell$-DF-2b	0.16 ± 0.05	$<0.001$	0.063 ± 0.013	0.23 ± 0.05	$<0.001$	0.64 ± 0.09	1

## Systematic uncertainties

The normalisation of signal and background in each channel can be affected by several sources of systematic uncertainty. These are described in the following subsections.

### Luminosity

The uncertainty in the integrated luminosity in the 2015 dataset is 2.1%. It is derived, following a methodology similar to that detailed in Ref. [68], from a calibration of the luminosity scale using *x*–*y* beam-separation scans performed in August 2015. This systematic uncertainty is applied to all processes modelled using Monte Carlo simulations.

### Uncertainties associated with reconstructed objects

Uncertainties associated with the lepton selection arise from imperfect knowledge of the trigger, reconstruction, identification and isolation efficiencies, and lepton momentum scale and resolution [47–50, 69]. The uncertainty in the electron identification efficiency is the largest systematic uncertainty in the trilepton channel and among the most important ones in the tetralepton channel.

Uncertainties associated with the jet selection arise from the jet energy scale (JES), the JVT requirement and the jet energy resolution (JER). Their estimations are based on Run-1 data and checked with early Run-2 data. The JES and its uncertainty are derived by combining information from test-beam data, collision data and simulation [70]. JES uncertainty components arising from the in-situ calibration and the jet flavour composition are among the dominant uncertainties in the $2\mu$-SS and trilepton channels. The uncertainties in the JER and JVT have a significant effect at low jet $p_{\text {T}}$. The JER uncertainty results in the second largest uncertainty in the trilepton channel.

The efficiency of the flavour-tagging algorithm is measured for each jet flavour using control samples in data and in simulation. From these measurements, correction factors are defined to correct the tagging rates in the simulation. In the case of *b*-jets, correction factors and their uncertainties are estimated based on observed and simulated *b*-tagging rates in $t\bar{t}$ dilepton events [62]. In the case of *c*-jets, they are derived based on jets with identified $D^{*}$ mesons [71]. In the case of light-flavour jets, correction factors are derived using dijet events [71]. Sources of uncertainty affecting the *b*- and *c*-tagging efficiencies are considered as a function of jet $p_{\text {T}}$, including bin-to-bin correlations [62]. An additional uncertainty is assigned to account for the extrapolation of the *b*-tagging efficiency measurement from the $p_{\text {T}}$ region used to determine the scale factors to regions with higher $p_{\text {T}}$. For the efficiency to tag light-flavour jets, the dependence of the uncertainty on the jet $p_{\text {T}}$ and $\eta$ is considered. These systematic uncertainties are taken as uncorrelated between *b*-jets, *c*-jets, and light-flavour jets.

The treatment of the uncertainties associated with reconstructed objects is common to all three channels, and thus these are considered as correlated among different regions.

### Uncertainties in signal modelling

From the nominal MG5_aMC+Pythia  8 (A14 tune) configuration, two parameters are varied to investigate uncertainties from the modelling of the $t\bar{t} Z$ and $t\bar{t} W$ processes: the renormalisation ($\mu _\mathrm{R}$) and factorisation ($\mu _\mathrm{F}$) scales. A simultaneous variation of $\mu _\mathrm{R} = \mu _\mathrm{F}$ by factors 2.0 and 0.5 is performed. In addition, the effects of a set of variations in the tune parameters (A14 eigentune variations), sensitive to initial- and final-state radiation, multiple parton interactions and colour reconnection, are evaluated. Studies performed at particle level show that the largest impact comes from variations in initial-state radiation [26]. The systematic uncertainty due to the choice of generator for the $t\bar{t} Z$ and $t\bar{t} W$ signals is estimated by comparing the nominal sample with one generated with Sherpa v2.2. The Sherpa sample uses the LO matrix element with up to one (two) additional parton(s) included in the matrix element calculation for $t\bar{t} Z$ ($t\bar{t} W$) and merged with the Sherpa parton shower [72] using the ME+PS@LO prescription. The NNPDF3.0NLO PDF set is used in conjunction with a dedicated parton shower tune developed by the Sherpa authors. Signal modelling uncertainties are treated as correlated among channels.

### Uncertainties in background modelling

In the trilepton and $2\mu$-SS channels, the diboson background is dominated by *WZ* production, while *ZZ* production is dominant in the tetralepton channel. While the inclusive cross sections for these processes are known to better than 10%, they contribute to the background in these channels if additional *b*-jets and other jets are produced and thus have a significantly larger uncertainty.

In the trilepton and $2\mu$-SS channels, the normalisation of the *WZ* background is treated as a free parameter in the fit used to extract the $t\bar{t} Z$ and $t\bar{t} W$ signals. The uncertainty in the extrapolation of the *WZ* background estimate from the control region to signal regions with specific jet and *b*-tag multiplicities is evaluated by comparing predictions obtained by varying the renormalisation, factorisation and resummation scales used in MC generation. The uncertainties vary across the different regions and an overall uncertainty of $-50\%$ and $+100\%$ is used.

The normalisation of the *ZZ* background is treated as a free parameter in the fit used to extract the $t\bar{t} Z$ and $t\bar{t} W$ signals. In the tetralepton channel, several uncertainties in the *ZZ* background estimate are considered. They arise from the extrapolation from the 4$\ell$-ZZ-CR control region (corresponding to on-shell *ZZ* production) to the signal region (with off-shell *ZZ* background) and from the extrapolation from the control region without jets to the signal region with at least one jet. They are found to be 30 and 20%, respectively. An additional uncertainty of 10–30% is assigned to the normalisation of the heavy-flavour content of the *ZZ* background, based on a data-to-simulation comparison of events with one *Z* boson and additional jets and cross-checked with a comparison between different *ZZ* simulations [11].

The uncertainty in the $t\bar{t} H$ background is evaluated by varying the factorisation and renormalisation scales up and down by a factor of two with respect to the nominal value, $H_\mathrm{T}/2$, where $H_\mathrm{T}$ is defined as the scalar sum of the transverse masses $\sqrt{p_{\text {T}} ^2+m^2}$ of all final state particles.

For the $tZ$ background, an overall normalisation uncertainty of 50% is assumed. An additional uncertainty affecting the distribution of this background as a function of jet and *b*-jet multiplicity is evaluated by varying the factorisation and renormalisation scales, as well as the amount of radiation in the Perugia2012 parton-shower tune.

An uncertainty of $+10\%$ and $-22\%$ is assigned to the $tWZ$ background cross section. The uncertainty is asymmetric due to an alternative estimate of the interference effect between this process and the $t\bar{t} Z$ production. The shape uncertainty is evaluated by varying the factorisation and renormalisation scales up and down by a factor of two with respect to the nominal value $H_\mathrm{T}/2.$

For other prompt-lepton backgrounds, uncertainties of 20% are assigned to the normalisations of the *WH* and *ZH* processes, based on calculations from Ref. [73]. An uncertainty of 50% is considered for triboson and same-sign *WW* processes.

The fake-lepton background uncertainty is evaluated as follows. The uncertainty due to the matrix method is estimated by propagating the statistical uncertainty on the measurement of the fake-lepton efficiencies. Additionally, a 20% uncertainty is added to the subtracted charge-flip yields estimated as the difference between data-driven charge-flips and simulation, and the $E_{\text {T}}^{\text {miss}}$ requirement used to enhance the single-fake-lepton fraction is varied by $20\,\text {GeV}$. The main sources of fake muons are decays of light-flavour or heavy-flavour hadrons inside jets. For the $2\mu$-SS region, the flavour composition of the jets faking leptons is assumed to be unknown. To cover this uncertainty, the central values of the fake-lepton efficiencies extracted from the *b*-veto and the *b*-tag control regions are used, with the efficiency difference assigned as an extra uncertainty. For the tetralepton channel, fake-lepton systematic uncertainties are covered by the scale-factor uncertainties used to calibrate the simulated fake-lepton yield in the control regions. Within a fake-lepton estimation method, all systematic uncertainties are considered to be correlated among analysis channels and regions. Thus $2\mu$-SS and trilepton fake-lepton systematic uncertainties that use the matrix method are not correlated with the tetralepton systematic uncertainties. The expected uncertainties in the fake-lepton backgrounds relative to the total backgrounds vary in each channel and signal region: 50% for the $2\mu$-SS region, 25–50% for the trilepton channel and 5–10% for the tetralepton channel.

## Results

In order to extract the $t\bar{t} Z$ and $t\bar{t} W$ cross sections, nine signal regions ($2\mu$-SS, 3$\ell$-Z-1b4j, 3$\ell$-Z-2b3j, 3$\ell$-Z-2b4j, 3$\ell$-noZ-2b, 4$\ell$-DF-1b, 4$\ell$-DF-2b, 4$\ell$-SF-1b, 4$\ell$-SF-2b) and two control regions (3$\ell$-WZ-CR, 4$\ell$-ZZ-CR) are simultaneously fitted. The $2\mu$-SS signal region is particularly sensitive to $t\bar{t} W$, the 3$\ell$-noZ-2b signal region is sensitive to both, $t\bar{t} W$ and $t\bar{t} Z$, while all other signal regions aim at the determination of the $t\bar{t} Z$ cross section. The cross sections $\sigma _{t\bar{t} Z}$ and $\sigma _{t\bar{t} W}$ are determined using a binned maximum-likelihood fit to the numbers of events in these regions. The fit is based on the profile-likelihood technique, where systematic uncertainties are allowed to vary as nuisance parameters and take on their best-fit values. None of the uncertainties are found to be significantly constrained or pulled from their initial values. The calculation of confidence intervals and hypothesis testing is performed using a modified frequentist method as implemented in RooStats [74, 75].

**Fig. 7:**
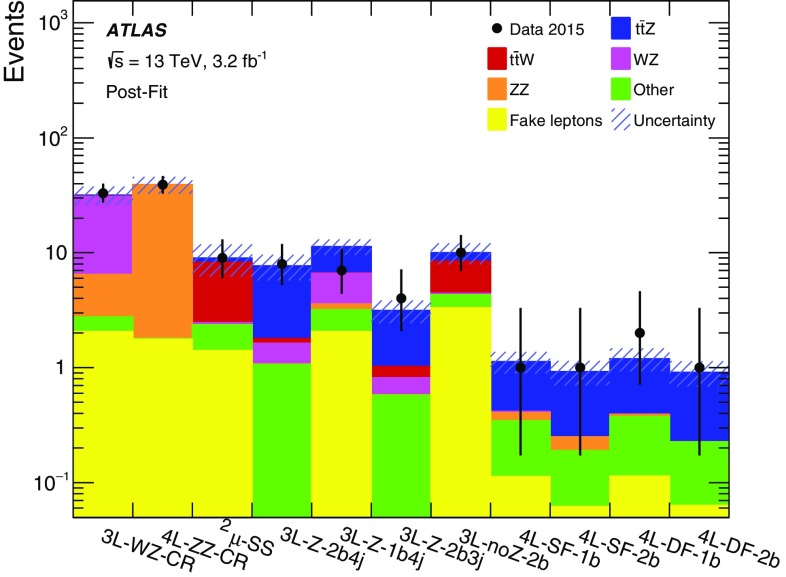
Expected yields after the fit compared to data for the fit to extract $\sigma _{t\bar{t} Z}$ and $\sigma _{t\bar{t} W}$ in the signal regions and in the control regions used to constrain the *WZ* and *ZZ* backgrounds. The ‘Other’ background summarises all other backgrounds described in Sect. 3. The shaded band represents the total uncertainty

A summary of the fit to all regions used to measure the $t\bar{t} Z$ and $t\bar{t} W$ production cross sections are shown in Fig. 7. The normalisation corrections for the *WZ* and *ZZ* backgrounds with respect to the Standard Model predictions are obtained from the fits as described in Sect. 5 and found to be compatible with unity: $1.11 \pm 0.30$ for the *WZ* background and $0.94 \pm 0.17$ for the *ZZ* background.

**Fig. 8:**
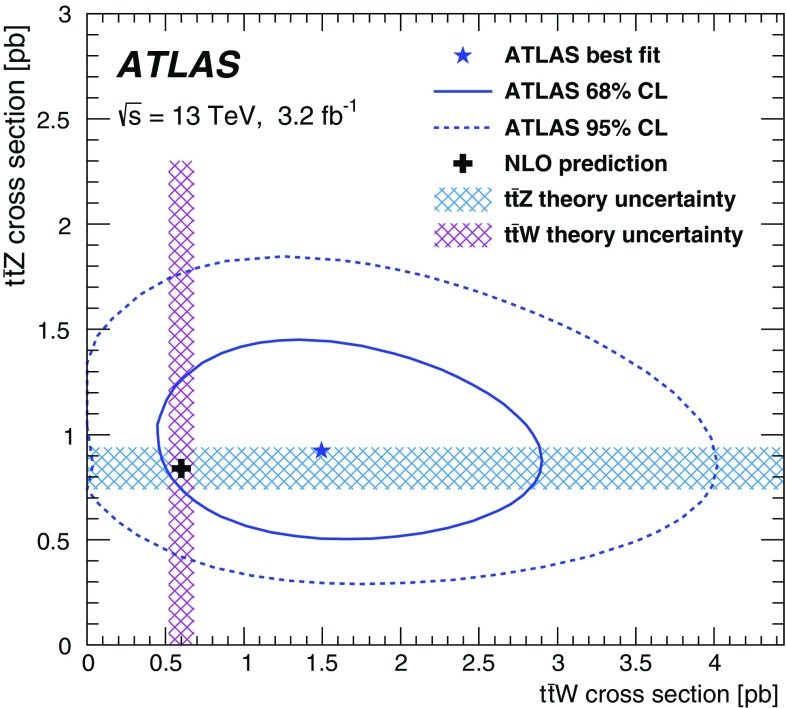
The result of the simultaneous fit to the $t\bar{t} Z$ and $t\bar{t} W$ cross sections along with the 68 and 95% confidence level (CL) contours. The *shaded areas* correspond to the theoretical uncertainties in the Standard Model predictions, and include renormalisation and factorisation scale uncertainties as well as PDF uncertainties including $\alpha _{\text {S}}$ variations

The results of the fit are $\sigma _{t\bar{t} Z} = 0.92 \pm 0.29 \;\text{(stat.) } \pm 0.10 \;\text{(syst.) }$ pb and $\sigma _{t\bar{t} W} = 1.50 \pm 0.72 \;\text{(stat.) } \pm 0.33 \;\text{(syst.) }$ pb with a correlation of $-0.13$ and are shown in Fig. 8. The fit yields significances of $3.9\sigma$ and $2.2\sigma$ over the background-only hypothesis for the $t\bar{t} Z$ and $t\bar{t} W$ processes, respectively. The expected significances are $3.4\sigma$ for $t\bar{t} Z$ and $1.0\sigma$ for $t\bar{t} W$ production. The significance values are computed using the asymptotic approximation described in Ref. [76]. In the two channels most sensitive to the $t\bar{t} W$ signal the observed relative number of events with two positively or two negatively charged leptons is compatible with expectation. In the 3$\ell$-noZ-2b channel the observed distribution of the number of events with a given amount of electrons and muons match expectation, as well.

Table 5 shows the leading and total uncertainties in the measured $t\bar{t} Z$ and $t\bar{t} W$ cross sections. In estimating the uncertainties for $t\bar{t} Z$ ($t\bar{t} W$), the cross section for $t\bar{t} W$ ($t\bar{t} Z$) is fixed to its Standard Model value. For both processes, the precision of the measurement is dominated by statistical uncertainties. For the $t\bar{t} Z$ determination, the different sources contribute with similar size to the total systematic uncertainty. For the $t\bar{t} W$ determination, the dominant systematic uncertainty source is the limited amount of data available for the estimation of the fake leptons.

**Table 5:** List of dominant and total uncertainties in the measured cross sections of the $t\bar{t} Z$ and $t\bar{t} W$ processes from the fit. All uncertainties are symmetrised

Uncertainty	$\sigma _{t\bar{t} Z} (\%)$	$\sigma _{t\bar{t} W} (\%)$
Luminosity	2.6	3.1
Reconstructed objects	8.3	9.3
Backgrounds from simulation	5.3	3.1
Fake leptons and charge misID	3.0	19
Signal modelling	2.3	4.2
Total systematic	11	22
Statistical	31	48
Total	32	53

## Conclusion

Measurements of the production cross sections of a top-quark pair in association with a *Z* or *W* boson using $3.2\,\text{ fb }^{-1}$ of data collected by the ATLAS detector in $\sqrt{s} = 13\,\text {TeV}$ *pp* collisions at the LHC are presented. Final states with either two same-charge muons, or three or four leptons are analysed. From a simultaneous fit to nine signal regions and two control regions, the $t\bar{t} Z$ and $t\bar{t} W$ production cross sections are determined to be $\sigma _{t\bar{t} Z} = 0.9 \pm 0.3$ pb and $\sigma _{t\bar{t} W} = 1.5 \pm 0.8$ pb. Both measurements are consistent with the NLO QCD theoretical calculations, $\sigma _{t\bar{t} Z} = 0.84 \pm 0.09\,\text {pb}$ and $\sigma _{t\bar{t} W} = 0.60 \pm 0.08\,\text {pb}$.

## Footnotes

1 In this paper, lepton is used to denote electron or muon, and prompt lepton is used to denote a lepton produced in a *Z* or *W* boson or $\tau$-lepton decay.

2 ATLAS uses a right-handed coordinate system with its origin at the nominal interaction point (IP) in the centre of the detector and the *z*-axis along the beam pipe. The *x*-axis points from the IP to the centre of the LHC ring, and the *y*-axis points upward. Cylindrical coordinates $(r,\phi )$ are used in the transverse plane, $\phi$ being the azimuthal angle around the *z*-axis. The pseudorapidity is defined in terms of the polar angle $\theta$ as $\eta =-\ln \tan (\theta /2)$.

3 A particle is considered stable if $c\tau \ge 1$ cm.

4 A primary vertex candidate is defined as a vertex with at least five associated tracks, consistent with the beam collision region. If more than one such vertex is found, the vertex candidate with the largest sum of squared transverse momenta of its associated tracks is taken as the primary vertex.
